# 12-O-Tetradecanoylphorbol-13-acetate increases cardiomyogenesis through PKC/ERK signaling

**DOI:** 10.1038/s41598-020-73074-4

**Published:** 2020-09-28

**Authors:** Katarzyna Anna Radaszkiewicz, Deborah Beckerová, Lucie Woloszczuková, Tomasz Witold Radaszkiewicz, Petra Lesáková, Olga Vondálová Blanářová, Lukáš Kubala, Petr Humpolíček, Jiří Pachernik

**Affiliations:** 1 Department of Experimental Biology, Faculty of Science, Masaryk University, Brno, Czech Republic; 2 Department of Free Radical Pathophysiology, Institute of Biophysics, Academy of Sciences of the Czech Republic, Brno, Czech Republic; 3 Centre of Polymer Systems and Faculty of Technology, Tomas Bata University in Zlin, 760 01 Zlin, Czech Republic

**Keywords:** Cell biology, Developmental biology, Stem cells

## Abstract

12-O-Tetradecanoylphorbol-13-acetate (TPA) is the most widely used diacylglycerol (DAG) mimetic agent and inducer of protein kinase C (PKC)-mediated cellular response in biomedical studies. TPA has been proposed as a pluripotent cell differentiation factor, but results obtained have been inconsistent. In the present study we show that TPA can be applied as a cardiomyogenesis-promoting factor for the differentiation of mouse embryonic stem (mES) cells in vitro. The mechanism of TPA action is mediated by the induction of extracellular signal-regulated kinase (ERK) activity and the subsequent phosphorylation of GATA4 transcription factor. Interestingly, general mitogens (FGF, EGF, VEGF and serum) or canonical WNT signalling did not mimic the effect of TPA. Moreover, on the basis of our results, we postulate that a TPA-sensitive population of cardiac progenitor cells exists at a certain time point (after days 6–8 of the differentiation protocol) and that the proposed treatment can be used to increase the multiplication of ES cell-derived cardiomyocytes.

## Introduction

Pluripotent stem (PS) cells represent a promising source of cardiomyocytes for cell-based therapies and a model for cardiac disease^1^. The cardiomyogenic differentiation of PS cells is an intensively investigated and already relatively well-described process. There are a number of efficient protocols for cardiomyocyte generation from PS cells, mainly based on in vivo and in vitro heart development studies. The most efficient protocols employ various growth factors (GFs), such as WNT, BMP, and FGF, to reinforce mesoderm and/or cardiac mesoderm differentiation during the initial steps. In the following phase, cardiac specification and maturation are driven by Dickkopf and Noggin—antagonists of the WNT and BMP pathways, respectively. Also, various well-characterised small-molecule compounds can be employed to imitate cellular responses to naturally occurring protein ligands involved in the aforementioned signalling pathways^2–4^. 12-O-Tetradecanoylphorbol-13-acetate (TPA) is a very potent small-molecule compound. It is standardly used in biomedical research as an activator of the protein kinase C (PKC) family, a broad group of serine/threonine kinases^5,6^. PKC signalling mediates a number of cellular processes, including the regulation of cell proliferation, differentiation, and migration, etc.^7–9^. Members of the PKC family are divided into three subfamilies on the basis of their mechanisms of activation. Classical PKCs require diacylglycerol (DAG), Ca^2+^ and acidic membrane phospholipids. Novel PKCs rely on DAG and acidic phospholipids. Atypical PKCs are reliant upon acidic phospholipids only. TPA mimics the action of DAG; therefore, it is involved in the activation of classical and novel PKCs^10^. TPA also triggers certain MAP kinase pathways and their downstream signalling events^11^.

In this study, we analysed the effect of TPA on cardiomyocyte formation in mouse embryonic stem (ES) cells. It was demonstrated that particular PKCs are associated with the regulation of cardiomyogenesis^12^. A study by Sachinidis et al*.* showed the inhibition of cardiomyocyte development after the TPA treatment of mES cells^13^. On the other hand, Feng et al*.* indicated that TPA induces the formation of endodermal lineage in human ES cells through PKC^14^, which can promote cardiac formation^15^. We showed that TPA treatment stimulates cardiomyogenesis in differentiating mES cells. This treatment increased the number of cardiomyocytes by approximately 5 times compared to the standard protocol of differentiation, which is based on spontaneous cardiomyogenesis induced by the formation of embryonic bodies. Surprisingly, the observed effect was limited to the specific time window at around Day 6 of differentiation. Thus, we suggest that stimulation by TPA targets the population of cardio-mesodermal progenitors. However, the accumulation of cellular response to prolonged treatment was not present. Various hypotheses about TPA’s mode of action were evaluated, including ERK and canonical WNT signalling cascades. In conclusion, our results show that the activation of PKC by TPA and subsequent stimulation of ERK at the specific time point increases the formation of cardiomyocytes in differentiating ES cells.

## Materials and methods

### Cell culture and cardiomyocyte differentiation

The undifferentiated mouse ES D3 line and R1 cell line and its subclones HG8, NK4 and TfR1 were adapted to feeder-free culture and routinely cultivated, as described previously^16,17^. R1_MHC-neor/pGK-hygro ES cells (HG8 cell line), carrying the *Myh6* promoter regulating the expression of neomycin phosphotransferase, were prepared by the transfection of R1 cells by MHC-neor/pGK-hygro plasmid (kindly provided by Dr. Loren J. Field, Krannert Institute of Cardiology, Indianapolis, US). R1_NKX2.5-GFP ES cells (clone NK4) carrying the *Nkx2.5* promoter-GFP reporter were prepared by the transfection of R1 cells with NKX2-5-Emerald GFP BAC reporter^18^. TfR1 ES cells were prepared by the transfection of R1 cells with Super8X TOPflash construct^19^. Cells were cultivated in DMEM media supplemented with 15% FBS, 100 IU/ml penicillin, 0.1 mg/ml streptomycin, and 1 × non-essential amino acid (all from Gibco-Invitrogen) and 0.05 mM β-mercaptoethanol (Sigma), supplemented with 1,000 U/ml of leukemia inhibitory factor (LIF, Chemicon), here referred to as complete ES medium. Cardiomyogenic differentiation was induced by the seeding of ES cells to form embryoid bodies (EBs). EB formation was induced by ES cell cultivation (2 × 10^5^ cells per ml) on bacteriological dishes coated with 1% agar in complete ES medium without LIF or by the hanging drop method (each EB was generated from 400 cells). In the next step, 5-day-old EBs were transferred onto adherent tissue culture dishes and cultivated in DMEM-F12 (1:1) supplemented with insulin, transferrin, selenium (ITS, Gibco-Invitrogene), and antibiotics (as above), i.e. the serum-free medium. The cell culture medium was replaced every 2 days. Selected intracellular signalling pathways were inhibited by the following drugs: CHIR99021 (inhibitor of GSK3 kinase), Go6976 (PKC inhibitor), Go6983 (PKC inhibitor), Chelerythrine chloride (PKC inhibitor), UO126 (inhibitor of MEK/ERK), and PD184352 (an inhibitor of MEK/ERK) (all from Sigma-Aldrich). In addition, recombinant WNT3A (R&D Systems) or WNT3A-conditioned medium were used for canonical Wnt pathway activation^20^ and FGF, EGF and VEGF (all Peprotech) for MEK/ERK pathway activation. TPA (P1585, Sigma-Aldrich) was used for PKC activation.

### Counting of cardiomyocytes

In each experiment the same initial number of ES cells were used, and each sample was derived from the same number of EBs. The relative number of cardiomyocytes in differentiating ES cell cultures was determined using the HG8 cell line as described before^16^. Briefly, cells were differentiated as mentioned above and on Day 14 and/or 20, MYH6-positive cardiomyocytes were selected by G418 antibiotic for six days. Estimation of the relative number of viable cells after antibiotic selection was performed by ATP quantification in whole cell lysates.

The direct counting of cardiomyocytes in differentiating ES cell cultures was performed using R1 cells as described before^16^. Briefly, EBs were seeded individually onto 24-well plates. On day 20, EBs were washed with phosphate buffered saline (PBS), incubated in a 0.3% solution of Collagenase II (Gibco) in DMEM media without serum for 20 min, and then incubated in trypsin (0.25% in PBS-EDTA, Gibco) for 5 min. Enzymes were inactivated by washing the cells with ES medium. Cells derived from each EB were seeded individually onto gelatinised cell culture wells. The next day, cells were washed with PBS, fixed with 4% formaldehyde, permeabilised by a 0.1% Triton X-100 solution in PBS, and blocked in 1% bovine serum albumin in PBS. Then, cells were incubated overnight at 4 °C with anti-cardiomyocyte heavy myosine antibody (anti-MHC, clone MF20, kindly provided by Dr. Donald Fischman, Developmental Studies Hybridoma Bank, Iowa City, IA, USA). The following day, cells were incubated with anti-mouse IgG conjugate Alexa488 (Invitrogen) for 1 h at RT. Nuclei were counterstained with DAPI (1 mg/l). Subsequently the number of cardiomyocytes per single EB was calculated manually using an Olympus IX-51 inverted fluorescence microscope (Olympus). Representative images were acquired using an Olympus IX-51 inverted fluorescence microscope (Olympus).

The percentage of cardiomyocytes in differentiating R1 and D3 ES cell cultures was determined by flow cytometry. Briefly, 20-day-old EBs were dissociated, as mentioned above. Cells were fixed with 4% formaldehyde (20 min/4 °C), permeabilised by a 0.1% Triton X-100 solution in PBS (10 min/RT), and blocked in 1% BSA in PBS (30 min/RT). Then, cells were stained with anti-MHC antibody (1 h/RT), followed by staining with anti-mouse IgG conjugate Alexa488 (1 h/RT). Simultaneously, negative controls—non-stained cells and cells stained only by secondary antibody—were prepared. Data were acquired with BD FACSVerse (BD Biosciences). Results were analyzed using NovoCyte software (ACEA Biosciences).

### Analysis of gene expression by qRT-PCR

Total RNA was extracted by RNeasy Mini Kit (Qiagen). Complementary DNA was synthesised using RevertAid Reverse Transcriptase reverse transcriptase kit (Thermo Fisher Scientific) following the manufacturer’s instructions. qRT-PCR was performed in a Roche Light-cycler using the following program: 95 °C for 10 min followed by 45 cycles of 95 °C for 10 s, 60 °C for 30 s, and 72 °C for 1 s, with data acquisition. Results are expressed as the fold difference between the target gene and the reference gene, determined by the relative quantification 2−ΔCq method. The levels of cardiac transcripts were determined using a LightCycler Probes Master with UniversalProbe library probes (all from Roche, Germany) according to the manufacturer’s instructions. Ribosomal protein L13A (*Rpl13a*) and *Hprt* were used as reference genes; primer sequences and probes are listed in Table 1.

**Table 1:** Probes and sequences of primers used in quantitative RT-PCR.

Gene of interest	Forward primer 5′ → 3′	Reverse primer 5′ → 3′	UPL probe no
*Hprt*	tcctcctcagaccgctttt	cctggttcatcatcgctaatc	#95
*Rpl13a*	ccctccaccctatgacaaga	gccccaggtaagcaaactt	#108
*Nkx2.5*	gacgtagcctggtgtctcg	gtgtggaatccgtcgaaagt	#53
*Myh6*	cgcatcaaggagctcacc	cctgcagccgcattaagt	#6
*Myh7*	cgcatcaaggagctcacc	ctgcagccgcagtaggtt	#6
*Myl2*	gtttgagcagacccagatcc	ttgtcgatgaagccgtctct	#93
*Myl7*	cccatcaacttcaccgtctt	aacatgcggaaggcactc	#7
*Mef2c*	accccaatcttctgccact	gatctccgcccatcagac	#6
*Gata4*	ggaagacaccccaatctcg	catggccccacaattgac	#13
*T*	actggtctagcctcggagtg	ttgctcacagaccagagactg	#27
*Mesp1*	acccatcgttcctgtacgc	gcatgtcgctgctgaagag	#89

### Western-blot analysis

Cells were directly lysed in 100 mM Tris/HCl (pH 6.8), 20% glycerol, 1% SDS, 0.01% bromophenol blue, and 1% 2-mercaptoethanol. Western blotting was performed according to the manufacturer's instructions with minor modifications [SDS-PAGE run on 110 V, transfer onto PVDF membrane for 1 h on 100 V (BIO-RAD)]. Membranes were blocked in 5% non-fat dry milk solution in TBS-T for 30 min and subsequently incubated overnight at 4 °C with the following primary antibodies: p-ERK1/2 (CS-4370S, Cell Signaling)^21^, ERK1/2 (CS-4695S, Cell Signaling)^21^, EGR1 (CS-4154, Cell Signaling)^21^, p-GATA4 (ab5245, Abcam)^22^, GATA4 (SC-25310, Santa Cruz Biotechnology)^23^, p-PKC (CS-9371, Cell Signaling)^24^, GFP ([3H9], Chromotek)^25^, β-Actin (CS-4970, Cell Signaling)^26^ and anti-cardiomyocyte myosine heavy chain antibody (anti-MHC, clone MF20)^27^. Next, membranes were washed in TBS-T and incubated with HRP-conjugated secondary antibodies (Sigma-Aldrich). Immunoreactive bands were detected using ECL detection reagent kit (Merck-Millipore) and the FusionSL chemiluminescence documentation system (Vilber-Lourmat). Western blot signals shown in the manuscript are present along with unprocessed and uncropped western blot images with molecular mass markers in the Supplementary Figures 13, 15, 17, 18, and 19. Western blot signals were obtained from membranes cut accordingly to the detected protein’s molecular size and incubated with primary and secondary antibodies alone to ascertain specific detection. This technique allows probing for various proteins using one membrane, but precludes the presence of full membranes. Therefore, the validation of signal specificity was performed using solvent- and TPA-treated samples (Supplementary Fig. 14, 16) and presented as full-size membranes. Red boxes in the Supplementary Figures 14 and 16 indicate the cropping strategy used in the manuscript. Results were quantified (Supplementary Fig. 9) by the densitometric analysis of Western blot bands using the Fiji distribution of ImageJ.

### TOPflash luciferase reporter assay

Tfr1 cells, the R1 subclone carrying the TOPflash luciferase reporter plasmid, were cultured and differentiated in the same manner as the maternal R1 ES cell line. For cell stimulation, recombinant WNT3A (R&D Systems), WNT3A conditioned media or control conditioned media^20^, TPA, and CHIR99021 (Sigma-Aldrich) were added 12 h before harvesting. Luciferase assay kit (Promega, USA) was used according to the manufacturer's instructions for the evaluation of luciferase activity. Relative luciferase units were measured on a Chameleon V luminometer (Hidex, Finland) and normalized to the cell mass.

### Measurement of reactive oxygen species induced by TPA

Human HL-60 cells were grown in RPMI 1640 (LM-R1640, Biosera) supplemented with 10% FBS and 100 IU/ml penicillin, and 0.1 mg/ml streptomycin at 37 °C in a 5% CO_2_ atmosphere. To differentiate HL60 cells into neutrophil-like cells, 1.3% DMSO was added to the medium for 5–7 days, as previously described^28,29^. The measurement of ROS was performed as reported before^29,30^. Briefly, cells were washed twice with serum-free and phenol-free medium. The reaction mixture consisted of 2.5 × 10^5^ cells, PKC/ERK inhibitor, 2 mM luminol (Sigma-Aldrich), and TPA. A total reaction volume of 120 µL was adjusted with serum-free, phenol-free medium. Assays were run in duplicates. Chemiluminescence (CL) emission, expressed as relative light units, was recorded continuously for 90 min at RT on a Chameleon V luminometer (Hidex, Finland). The results of the CL reaction are presented as total ROS production (manifested by luminescence measurements) in dependence on time.

### Isolation of mouse hearts

CD1 mice were maintained and bred under standard conditions and were used in accordance with the European Community Guidelines of accepted principles for the use of experimental animals. Mouse hearts were isolated according to an experimental protocol that was approved by the National and Institutional Ethics Committee (protocol MSMT-18110/2017-5). Embryonic hearts were collected from E8, E12, E13, E18 embryos isolated from sacrificed pregnant mice. Neonatal hearts were excised from newborn mice, whereas adult hearts were derived from male 2–4-months-old mice. Isolated hearts were kept on ice in cold PBS during subsequent manipulations. Whole hearts and separated atria and ventricles were immediately frozen. Subsequently, tissue was dissociated in lysis buffer (Qiagen) by tissue grinding pestle (Serva) and samples were processed as described in paragraph 2.3. Each sample of embryonic heart was formed from a pool of 4 hearts; samples of neonatal and adult hearts and their parts were formed from single hearts.

### Statistical analysis

Data are expressed as the mean ± SEM. Statistical analysis was assessed by one-way analysis of variance ANOVA, and Bonferroni’s Multiple Comparison post-test or t-test values of *P* < 0.05 were considered statistically significant.

## Results

### TPA increases cardiomyocyte formation in mES cells

**Figure 1:**
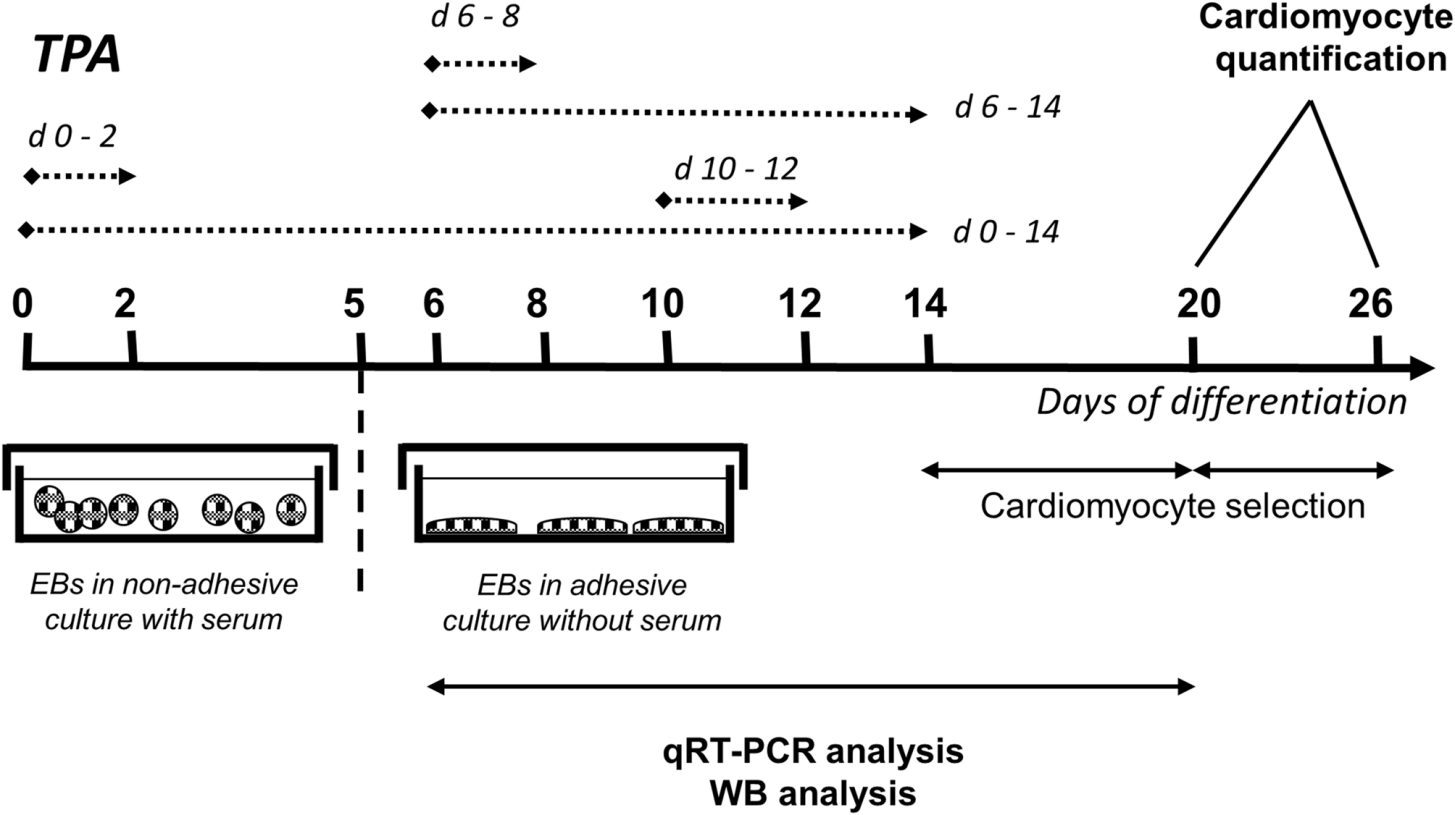
Experimental setup employed for comparison of the cardiomyogenic differentiation of ES cells exposed to different TPA treatment. Cells were treated with 1 µM of TPA on Days 0–2 (d 0–2), Days 0–14 (d 0–14), Days 6–8 (d 6–8), Days 6–14 (d 6–14), and Days 10–12 (d 10–12) of differentiation. The number of cardiomyocytes was calculated on Days 20 and 26. Additionally, cardiomyocyte markers were analysed between Day 7 and Day 20 by qRT-PCR and Western blot method.

**Figure 2:**
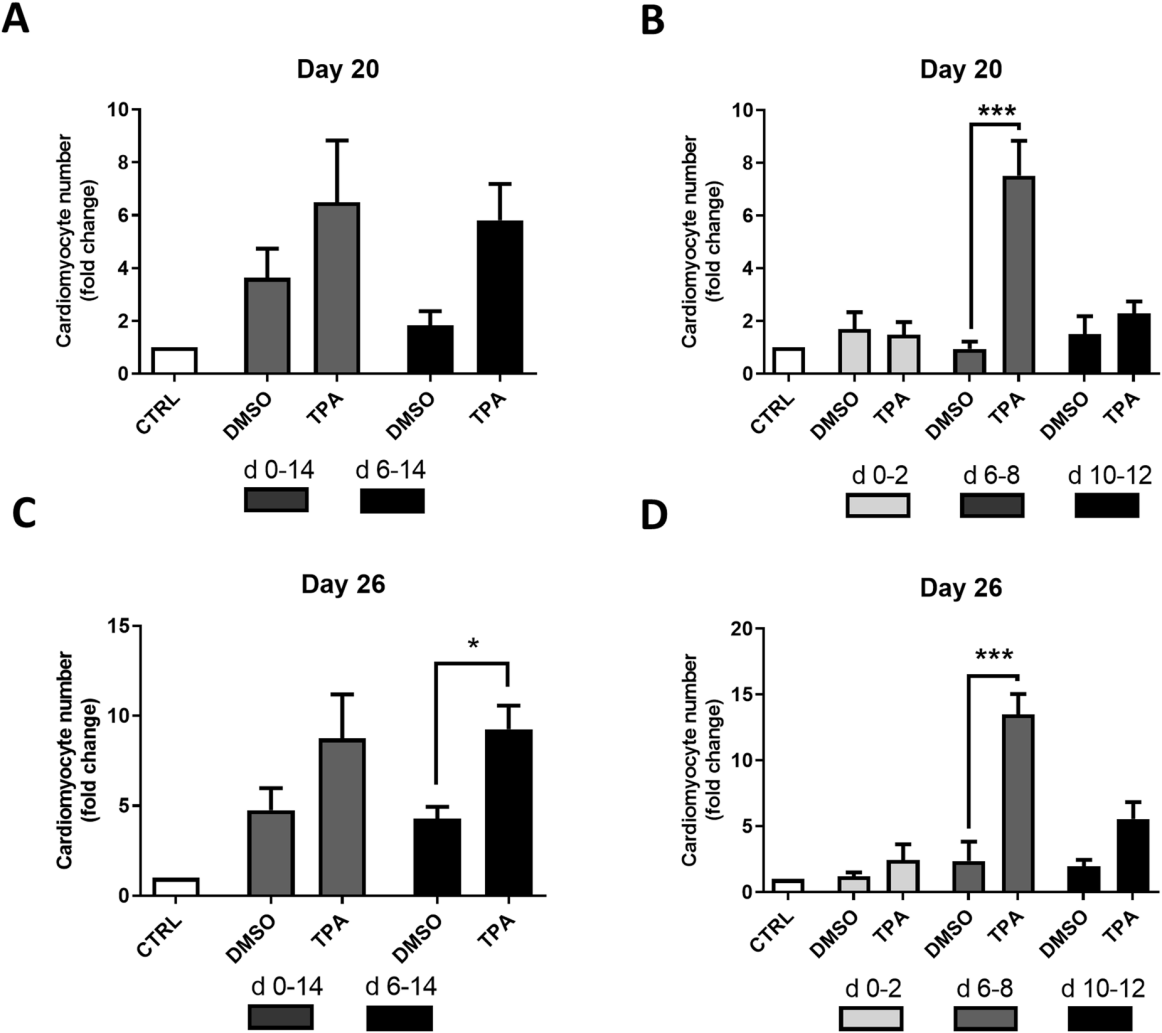
The effect of TPA treatment on the number of derived cardiomyocytes—indirect quantification. The number of cardiomyocytes was determined in HG8 cells treated with 0.1% DMSO and 1 µM TPA in different time windows (see Fig. 1). The selection of cardiomyocytes began on Day 14 and measurements were performed on Day 20 of differentiation (**A**, **B**). Selection of cardiomyocytes began on Day 20 and measurements were performed on Day 26 of differentiation (**C**, **D**). Data are presented as mean ± SEM, n ≥ 4. Statistical significance was determined by ANOVA with post hoc Bonferroni's Multiple Comparison test; *P < 0.05; **P < 0.01; ***P < 0.001.

**Figure 3:**
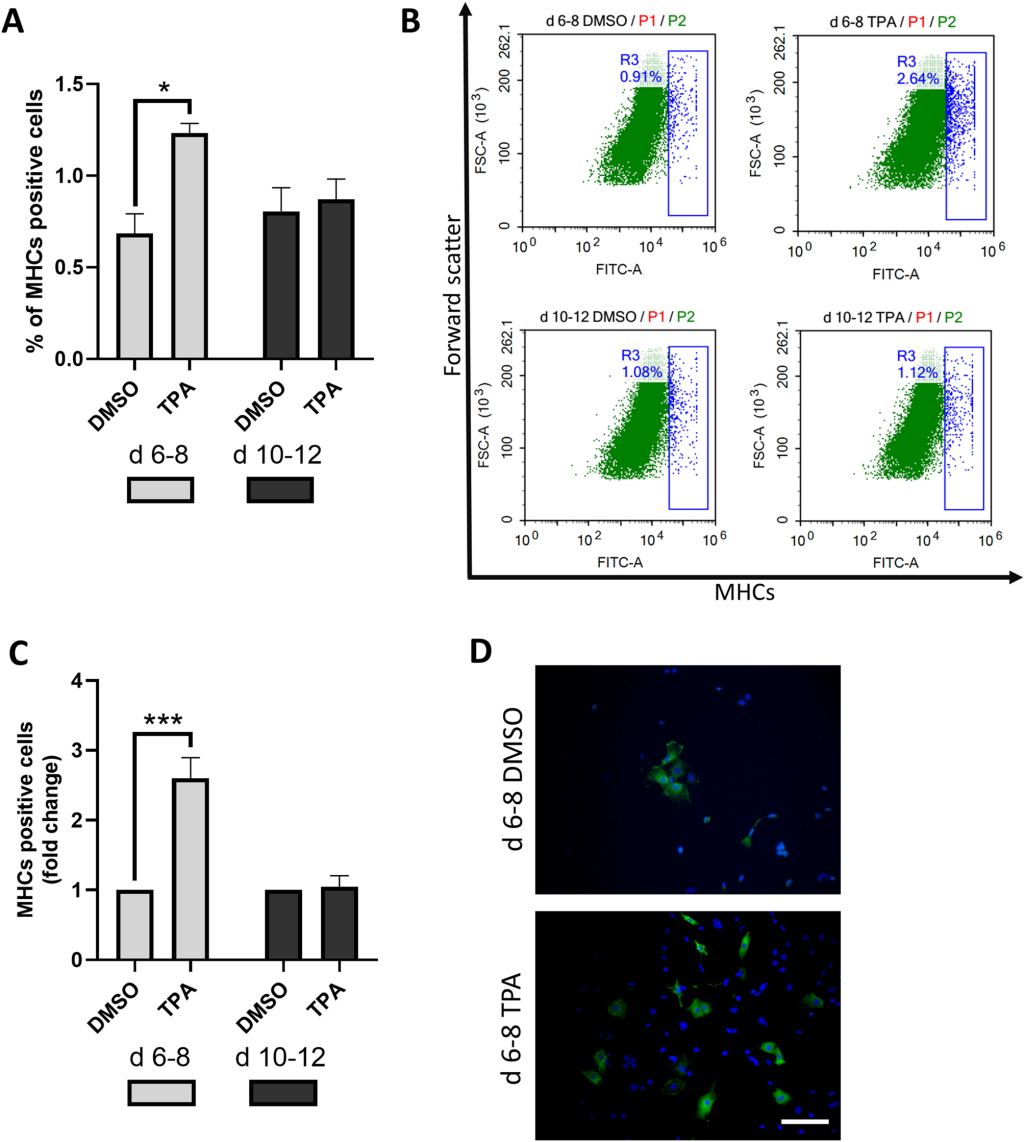
The effect of TPA treatment on the number of derived cardiomyocytes—direct quantification. R1 cells were treated with 1 µM of TPA on Days 6–8 (d 6–8) Days 10–12 (d 10–12) of differentiation. Flow cytometric quantification of myosine heavy chains (MHCs) was performed on Day 20. Percentage of MHCs positive cells (**A**). Representative plots; gate R3 shows the percentage of MHC-positive cells (see Supplementary Fig. 2 for cell gating strategy) (**B**). Direct counting of MHC-positive cells by immunofluorescence staining. The number of MHC-positive cells per one embryoid body was calculated (**C**). Representative photos of cardiomyocytes (**D**). Red signal (MHCs), blue signal (DAPI, nuclear stain). Scale bar, 50 µm. Data are presented as mean ± SEM, n ≥ 3. Statistical significance was determined by ANOVA with post hoc Bonferroni's Multiple Comparison test; * *P* < 0.05; **P < 0.01; ***P < 0.001.

Differentiating mES cells were treated with 1 µM TPA at various time intervals (1 µM TPA had the most evident effect in the preliminary tested concentration range of 0.01–2 µM; Supplementary Fig. 1 demonstrates the effect of 0.1 and 1 µM TPA). The number of cardiomyocytes was evaluated on Days 20 and 26 (Fig. 1). First, cells were exposed to TPA continuously from Day 0 to Day 14 and from Day 6 to Day 14. As a result, we obtained an approximately 3-times-higher yield of cardiomyocytes on Days 20 and 26 of differentiation for the treatment interval from Day 6 to Day 14 (Fig. 2A,C). Then, we examined whether the same effect could be achieved for other time periods (Day 0–Day 2, Day 6–Day 8, and Day 10–Day 12). Interestingly, the most pronounced result was obtained when the cells were exposed to TPA between Days 6 and 8 of differentiation (Fig. 2B,D). To further characterize the number of developing cardiomyocytes, flow cytometric analysis of myosine heavy chain (MHC)-positive cells was employed. Only single cell populations without debris were taken into analysis and appropriate negative controls were used to adjust the gating (Supplementary Fig. 2A–A’’; 3A–A’’). We observed that upon TPA treatment from Day 6 to Day 8 the number of MHC-positive cells increased approximately two times, whereas TPA exposition from Day 10 to Day 12 had no effect (Fig. 3A,B and Supplementary Fig. 3B,C). Because flow cytometry analysis gives information only about the relative number of cardiomyocytes, we also directly calculated the number of formed cardiomyocytes per single EB. In accordance with previous data, the treatment interval from Day 6 to Day 8 had the most prominent effect on cardiomyogenesis (Fig. 3C,D). To avoid potential false-positive results caused by the presence of DMSO, a solvent of TPA, an additional control was added to our experimental scheme. DMSO was found to have no effect on cardiomyogenesis.

### TPA stimulates cardiac mesoderm and promotes cardiomyogenesis

**Figure 4:**
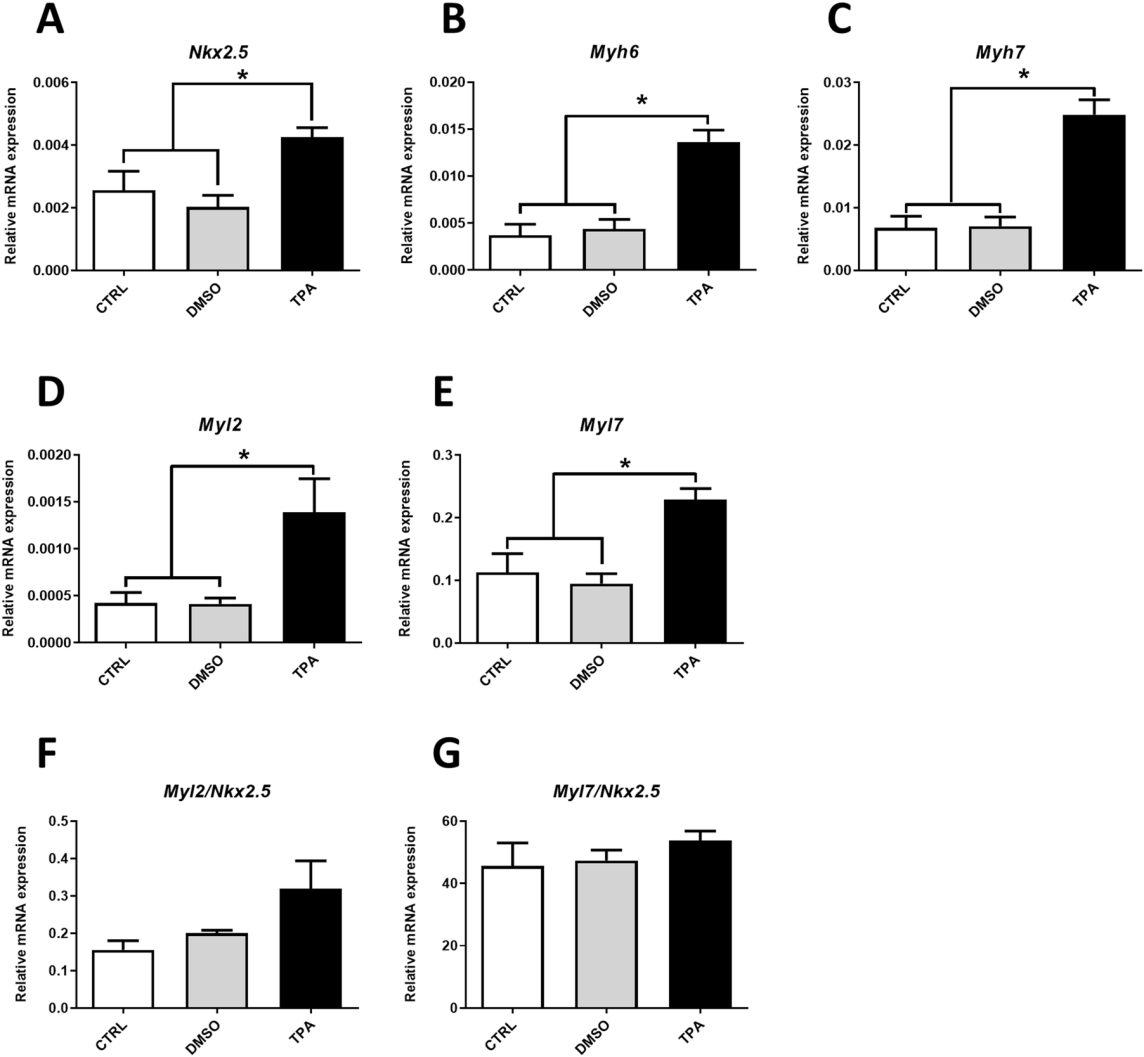
The effect of TPA treatment on the expression of cardiomyocyte specific transcripts. Relative mRNA levels in R1 cells differentiated for 20 days and treated with 0.1% DMSO and 1 µM TPA between Day 6 and Day 8 of differentiation, determined by qRT-PCR normalised to the mean expression of *Hprt* and *Rpl13a* genes (**A**–**E**) or normalised to the *Nkx2.5* gene (**F**, **G**). Data are presented as mean ± SEM, n ≥ 4. Statistical significance was determined by ANOVA with post hoc Bonferroni's Multiple Comparison test; *P < 0.05; **P < 0.01; ***P < 0.001.

For further evaluation of the TPA-mediated effect on cardiomyogenesis, the expressions of cardiomyocyte-specific genes (*Nkx2.5*, *Myh6*, *Myh7*, *Myl2*, *Myl7*) were determined on Day 20 of differentiation by qRT-PCR. Cells treated with TPA (time window 6–8D) exhibited higher expressions of the aforementioned transcripts compared to controls (Fig. 4A–E and Supplementary Fig. 4A–E).

We also calculated the levels of *Myl2* (ventricular cardiomyocyte isoform) and *Myl7* (atrial cardiomyocyte isoform) gene transcripts, which are frequently used for description of the ventricular vs. atrial specification of ESC derived cardiomyocytes (Supplementary Fig. 5C,D)^31,32^. Their expression was normalised to the expression of transcription factor *Nkx2.5* in order to elucidate the phenotype of cardiomyocytes. In contrast to myosin gene expression, the *Nkx2.5* transcript level is relatively stable in cardiac precursor cells and cardiomyocytes. Moreover, it is independent of their phenotype and maturation (Supplementary Fig. 5)^33,34^. Thus, *Nkx2.5* transcript can be used as a reference gene for cardiomyocytes within mixed cell populations such as differentiating ES cells. The expression of *Myls* was the same for all analysed groups (Fig. 4F,G and Supplementary Fig. 4F,G). Therefore, we can infer that TPA does not induce a change of cell phenotype.

**Figure 5:**
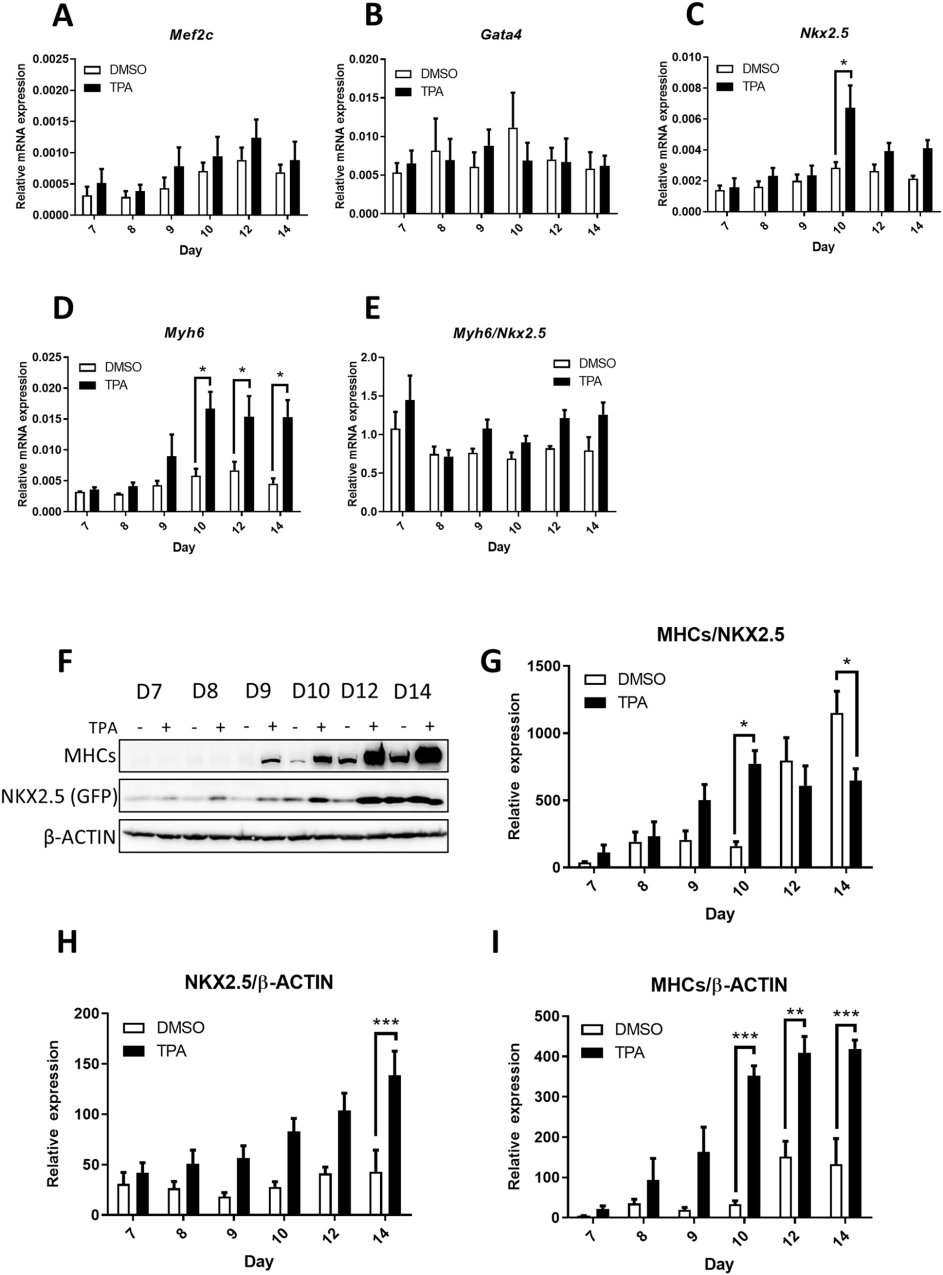
The TPA-mediated effect on the dynamics of the expression of cardiomyocyte specific transcripts at various time points. Relative mRNA levels of cardiac markers analysed at different time points of R1 cell differentiation determined by qRT-PCR, normalised to the mean expression of *Hprt* and *Rpl13a* genes (**A**–**D**) or normalised to *Nkx2.5* gene expression (**E**). Western blot analysis of myosine heavy chains (MHCs) and NKX2.5 level (as level of GFP) at different time points of R1 cell (subclone NK4, see “Materials and methods”) differentiation (**F**). Ratio of MHC/NKX2.5 (**G**) and relative level of NKX2.5 (as level of GFP; **H**) and MHC (**I**) to β-Actin determined by densitometric analysis. Data are presented as mean ± SEM, n ≥ 4. Statistical significance was determined by ANOVA with post hoc Bonferroni's Multiple Comparison test; *P < 0.05; **P < 0.01; ***P < 0.001.

Next, we analysed the expressions of cardiac mesoderm markers *Mef2c* (myocyte enhancer factor)^35^ and *Gata4*^36^, and genes characteristic of cardiomyocytes, namely *Nkx2.5* and *Myh6*, at different time points after TPA treatment. TPA did not affect *Mef2c* or *Gata4* expression (Fig. 5A,B). However, levels of *Nkx2.5* and *Myh6* transcripts were significantly increased—*Nkx2.5* on Day 10 only, while *Myh6* was increased on Days 10, 12, 14 of differentiation (Fig. 5C,D). The expression of *Myh6* transcript normalised to *Nkx2.5* transcript was the same at all determined time points (Fig. 5E). This suggests that cardiomyocytes underwent multiplication rather than maturation.

To verify the effect of TPA treatment also on the levels of cardiomyocyte-specific proteins, we performed Western blot analysis. Myosine heavy chains (MHCs) and NKX2.5 signals (determined as the level of GFP, the expression of which is driven by *Nkx2.5* promotor) increased proportionally upon TPA treatment (Fig. 5F,H,I). The level of MHCs was significantly augmented from Day 10 of differentiation, whereas NKX2.5 protein expression escalated on Day 14, which indicates that TPA promotes cardiomyogenesis (Fig. 5H,I). To determine the maturation status of cardiomyocytes, we normalised MHCs to the level of NKX2.5 (Fig. 5G). The MHCs/NKX2.5 ratio for TPA was significantly increased on Day 10 of differentiation, which could suggest the more progressed maturation status of cardiomyocytes. Interestingly, on Day 14, the ratio decreased significantly. Taken together, these data reveal that TPA induces the multiplication of cardiomyocyte progenitors, rather than their maturation. To support our hypothesis further, the employed cardiac differentiation protocol was characterized by means of gene expression analysis at different time points. The mesoderm marker *T*^37,38^ and the cardiac mesoderm marker *Mesp1*^38–40^ became highly expressed from Day 3, peaking on Day 5 (Supplementary Fig. 6A,B). Later, the cardiomyocyte marker *Nkx2.5*^32,33^ was upregulated from Day 5 and peaked on Day 15 (Supplementary Fig. 6C). Finally, cardiomyocyte myofilament gene *Myh6*^31,32^ peaked on Day 15 of differentiation (Supplementary Fig. 6D). On the basis of these observations, we conclude that the population targeted by TPA corresponds to early cardio-mesoderm progenitors.

### Role of the ERK pathway in cardiomyogenesis through TPA-induced PKC signalling

**Figure 6:**
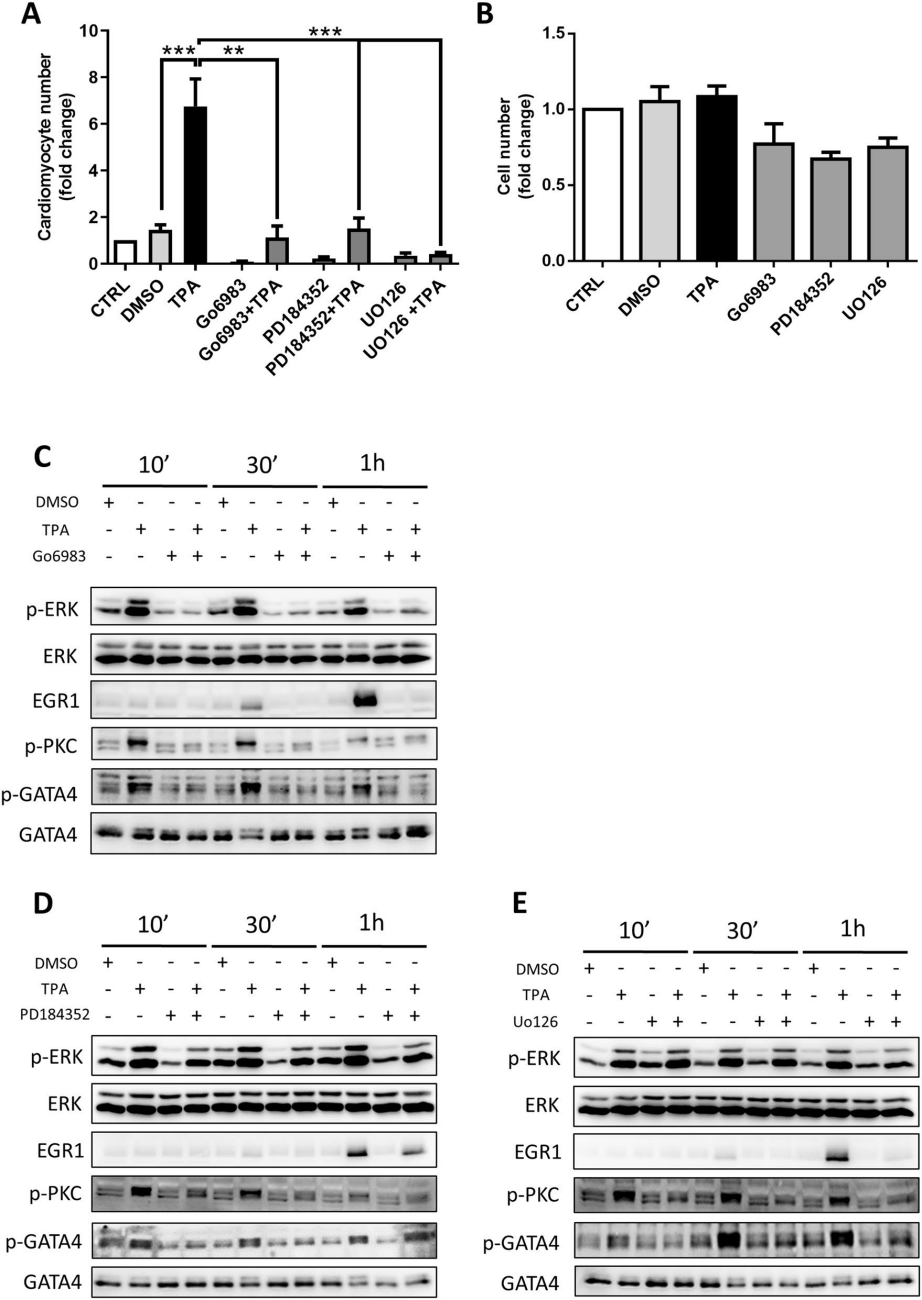
The role of PKC and ERK signalling pathways in TPA-induced cardiomyogenesis. The number of cardiomyocytes was determined after the treatment of differentiated HG8 cells with the following inhibitors from Day 6 to Day 8: 1 µM Go6983, 1 µM PD184352, and 5 µM UO126 in combination with 1 µM TPA and 0.1% DMSO as solvent control. The selection of cardiomyocytes began on Day 14 and measurements were performed on Day 20 of differentiation (**A**). Fold of change in cell number for the non-selected population on Day 14 of differentiation after treatments corresponding to “A” (**B**). Western blot analysis of the phosphorylation status of PKC, ERK, and GATA4 and the level of EGR1 after the treatment of R1 cell-derived 6-day-old embryoid bodies with 0.1% DMSO or 1 µM TPA and the aforementioned inhibitors in the indicated time intervals (**C**–**E**). Data are presented as mean ± SEM, n ≥ 3. Statistical significance was determined by ANOVA with post hoc Bonferroni's Multiple Comparison test; *P < 0.05; **P < 0.01; ***P < 0.001.

To identify the mechanism of TPA action in cardiomyocyte multiplication, we employed various inhibitors targeting PKC- and ERK-related signalling. We combined TPA treatments with the aforementioned inhibitors to elucidate their effect on cardiomyocyte formation. As shown in Fig. 6A, Go6983—an inhibitor of PKC—significantly reduced the number of TPA-induced cardiomyocytes. In contrast, other inhibitors of PKC protein—namely, Go6976 and chelerythrine chloride—did not influence the quantity of cardiomyocytes (Supplementary Fig. 7A). At the same time, both used ERK signalling inhibitors (PD184352 and UO126) decreased cardiomyocyte yield (Fig. 6A). To ascertain that the decline in cardiomyocyte number was not mediated by the general anti-mitogenic effect of PKC and ERK signalling inhibitors, we determined the total number of differentiated cells prior to cardiomyocyte selection under the tested conditions (on Day 14). Here, we did not detect any significant effect of TPA or inhibitors on the proliferation of these cells (Fig. 6B and Supplementary Fig. 7B). To confirm the role of TPA in PKC/ERK pathway activation in our model of cardiomyogenesis, we performed Western blot analysis of cells treated with TPA and inhibitors on Day 6 of differentiation. TPA induced the phosphorylation of PKC and ERK after 10 min, 30 min, and 1 h of exposure to TPA, thus showing their activation upon treatment^41,42^. Moreover, 1 h of TPA treatment increased the level of EGR1, the expression of which is propagated by active ERK signalling^43^ (Fig. 6C–E and Supplementary Fig. 7C,D; 8A–C; 9A–A’’, B–B’’, C–C’’).

Since TPA significantly activated PKC and ERK signalling, we investigated the effects of ERK activation and inhibition on the phosphorylation of GATA4. It is known that MAPKs cascades can modify the expression and activity of transcription factors by means of post-translational modifications such as phosphorylation^44^. Our data demonstrate that TPA induced the phosphorylation of GATA4 in our model of cardiomyogenesis (Fig. 6C,D,E and Supplementary Fig. 7C,D; 8A–C; 9D–D’’). For further evaluation of the mechanism of TPA action, we inhibited the PKC pathway by various inhibitors. Go6983 blocked the TPA-dependent phosphorylation of PKC and ERK and reduced the level of EGR1, and, as a consequence, impeded GATA4 phosphorylation (Fig. 6C and Supplementary Fig. 8A; 9A–D). In contrast, other inhibitors of PKC–Go6976 and chelerythrine chloride (CHEL), did not cause this effect (Supplementary Fig. 7C,D). To study downstream events of PKC signaling, we applied specific ERK inhibitors. Treatments by both UO126 and PD184352 abolished the phosphorylation of GATA4 (Fig. 6D,E and Supplementary Fig. 8B,C; 9A’–D’, A’’–D’’). Interestingly, these inhibitors decreased the phosphorylation of PKC to some extent (Supplementary Fig. 9C’,C’’).

Taking these findings together with the results of cardiomyocyte counting (Fig. 6A and Supplementary Fig. 7A), we suggest that the positive effect of TPA on cardiomyocyte differentiation is mediated through the activation of the ERK signalling pathway by PKC. Moreover, our data suggest that TPA stimulates cardiac mesoderm cells through the phosphorylation of GATA4 transcription factor.

## Discussion

Our work shows that TPA is able to enhance the cardiomyogenesis of mouse ES cells. TPA induces the multiplication of cardiomyocyte progenitors through ERK signalling cascades and the subsequent phosphorylation of GATA4, which leads to an approximately 5-times-higher number of formed cardiomyocytes. Interestingly, this effect is specific to a relatively short time window of the differentiation process and is not strengthened by prolonged treatment.

TPA is known as a potent inducer of multiple signalling pathways in a PKC-dependent manner^45^. TPA is frequently used in biomedical studies because of its ability to mimic diacylglycerol (DAG) and consequently activate classical and novel PKCs^10^. PKCs are generally expressed in all tissues during embryonal development^46^, but their isoforms differ in their tissue distributions and perform distinct biological functions^47^. Interestingly, it was shown that, depending on the stimulation, individual PKCs can play antagonistic roles in the same cells^48^. In addition, PKCs activated by TPA can also stimulate other signalling pathways—for instance MAPKs cascades, by the phosphorylation of downstream components^11^. MAPKs are serine-threonine kinases involved in numerous processes, including those controlling cell proliferation and differentiation^49^.

The role of PKCs and TPA in cardiomyogenesis in vitro is controversial and available data is contradictory. It was demonstrated that the downregulation of PKCβ, together with the upregulation of PKCε, are crucial for the formation of cardiomyocytes from ES cells^12,50,51^. In contrast, Ventura et al*.* showed that PKCε and PKCβ levels increase during the process of cardiomyogenesis and that the inhibition of classical/novel PKCs impairs the formation of cardiac cells^52^. However, Sachinidis et al*.* implied that the inhibition of PKC signalling improves cardiac formation, while TPA treatment decreases the yield of cardiomyocytes^13^. Interestingly, TPA can rescue cardiomyogenesis altered by the disruption of FGF1/FGFR signaling^53^. The reasons for such discrepancies are unclear. Possibly, they can be the result of the application of different cardiomyogenesis protocols, different timings of treatments, or the different complexities of the particular cardiomyogenesis evaluation methods employed.

The enhancement of cardiomyogenesis by TPA has not previously been recognized. We showed that TPA treatment and the activation of PKCs in a specific time window of differentiation strengthen cardiomygonesis.

Our results suggest that TPA-induced ERK1,2 activity and subsequent GATA4 phosphorylation is a key mechanism responsible for more pronounced cardiomyogenesis in mES cells. The extracellular signal-regulated kinases ERK1 and ERK2 are the best-characterised MAPKs, which also contribute to cardiac development^54^. TPA induced both PKC and ERK phosphorylation and expression of the ERK target gene, *Egr-1*^43^. Furthermore, inhibitors of ERK signalling diminished TPA-mediated activity, which confirms the important role of this pathway in cardiac formation. In contrast, the effect of PKC inhibitors on TPA activation was not consistent. Only one inhibitor, Go6983, efficiently blocked TPA action. The observed discrepancies can be explained by inhibitor specificity. Chelerythrine chloride was described as a potent PKC inhibitor^55^. However, latest reports show that chelerythrine does not inhibit either basal or TPA-induced PKC activity^56^. On the other hand, Go6976 was described as a strong inhibitor of PKCα and PKCβ isoforms, but not of novel PKC isozymes, which can also be activated by TPA^56^. Nevertheless, we cannot state which isoform is responsible for the observed TPA effect on cardiomyogenesis. To address the question of inhibitor specificity towards PKCs, we performed additional tests based on the measurement of oxidative burst in neutrophil-like cells (Supplementary Fig. 10A,B). TPA activates PKC, which in turn promotes NADPH-dependent superoxide production^57^. In our experimental model, Go6983 inhibited the generation of reactive oxygen (ROS) species mediated by TPA, whereas Go6976 and ERK inhibitors did not show any effect. Surprisingly, chelerythrine chloride worked similarly to Go6983. However, it was reported that the concentration of chelerythrine chloride which inhibits oxidative burst activated by TPA is not enough to inhibit the activity of PKC^58^. Taken together, our results clearly indicate that TPA promotes the activation of the ERK pathway by PKC induction, followed by the phosphorylation of GATA4. Interestingly, TPA treatment promoted the phosphorylation of GATA4, but did not have any impact on the overall level of GATA4 expression. GATA4 phosphorylation increases its activity by means of an increase in DNA binding or by promoting interactions with co-activators, which leads to the promotion of the cardiac gene program^44,59^.

The observed acceleration of cardiomyogenesis by TPA was limited to a relatively short time window (from Day 6 to Day 8). TPA treatment did not increase the level of *Nkx2.5* transcripts immediately, so we suggest that TPA affects a subpopulation of NKX2.5-negative cardiomyogenic progenitors. We postulate that TPA affects Brachyury T- or Mesp1-positive cells. *Brachyury* *T* and *Mesp1* are key genes regulating mesoderm and cardiomyogenic mesoderm formation, respectively^60–62^. In our experimental models, their expression peaked on day 5 (Supplementary Fig. 6A,B), the time when TPA shows the most pronounced effect, which confirms our hypothesis. Moreover, this effect seems to be specific to particular cells, because the overall mitogenic effect of TPA was not observed. Nevertheless, the identification of these cells will be the aim of further studies. Importantly, a recent study also showed that the activation of MAPK cascades was observed during a similar time window of cardiac differentiation (5–8 days)^23^.

To further characterise the TPA effect on ERK signalling, we tried to mimic its activity using growth factors. To this end, cells were treated with EGF, FGF2, VEGF, and serum. These factors are known to mediate mitogenic stimulation and activate ERK signalling in cells, which is also sensitive to PKC signalling cascade^45,63^. Nevertheless, none of these inducers exhibited the effect delivered by TPA (Supplementary Fig. 11). However, these results can be affected by the insufficient stimulation of cells within compact EBs. It is known that in contrast to TPA, big hydrophilic proteins such as GF have limited ability to cross multi-layered spheroids^5,64,65^.

Another pathway cross-reacting with TPA and PKCs is canonical WNT signalling. The formation of early cardiomyogenic mesoderm and its proliferation is regulated by the WNT/β-catenin pathway^66^. The phosphorylation of LRP6, a key component of this pathway, is mediated by the activation of various MAPKs, which can be induced through TPA treatment. Phosphorylated LRP6 protein then significantly reinforces the WNT-mediated signal^67^. We investigated whether TPA can strengthen WNT/β-catenin signalling and thus modify the dynamics of cardiomyogenesis. From Day 6 to Day 8, differentiating cells were treated with TPA, WNT3A, and the canonical WNT signalling mimetic CHIR99021, an inhibitor of GSK3 kinase. Similarly to previous results, TPA induced cardiomyocyte multiplication, whereas WNT3A and CHIR99021 had no effect (Supplementary Fig. 12). We did not observe any effect of TPA on canonical WNT signalling in our experiments, which shows that the observed cardiomyocyte multiplication was not WNT/β-catenin dependent.

In conclusion, we demonstrated that TPA can be used to reinforce cardiomyogenesis in mES cells. However, additional studies will be necessary to describe the mechanism of the TPA-induced multiplication of cardiac progenitors and to identify the target population of cardiomyocyte precursors.

## Supplementary information

Supplementary Figures.

## Data Availability

The data that support the findings of this study are available from the corresponding author (Jiří Pacherník) upon reasonable request.

## References

[1] Hartman ME, Dai DF, Laflamme MA (2016). Human pluripotent stem cells: prospects and challenges as a source of cardiomyocytes for in vitro modeling and cell-based cardiac repair. Adv. Drug Deliv. Rev..

[2] Noseda M, Peterkin T, Simoes FC, Patient R, Schneider MD (2011). Cardiopoietic factors extracellular signals for cardiac lineage commitment. Circ. Res..

[3] Batalov I, Feinberg AW (2015). Differentiation of cardiomyocytes from human pluripotent stem cells using monolayer culture. Biomark. Insights.

[4] Ichimura H, Shiba Y (2017). Recent progress using pluripotent stem cells for cardiac regenerative therapy. Circ. J..

[5] Goel G, Makkar HPS, Francis G, Becker K (2007). Phorbol esters: structure, biological activity, and toxicity in animals. Int. J. Toxicol..

[6] Mochly-Rosen D, Das K, Grimes KV (2012). Protein kinase C, an elusive therapeutic target?. Nat. Rev. Drug Discov..

[7] Barnett ME, Madgwick DK, Takemoto DJ (2007). Protein kinase C as a stress sensor. Cell. Signal..

[8] Papp H (2004). Opposite roles of protein kinase C isoforms in proliferation, differentiation, apoptosis, and tumorigenicity of human HaCaT keratinocytes. Cell. Mol. Life Sci..

[9] Fogh BS, Multhaupt HAB, Couchman JR (2014). Protein kinase C, focal adhesions and the regulation of cell migration. J. Histochem. Cytochem..

[10] Way KJ, Chou E, King GL (2000). Identification of PKC-isoform-specific biological actions using pharmacological approaches. Trends Pharmacol. Sci..

[11] Schönwasser DC, Marais RM, Marshall CJ, Parker PJ (1998). Activation of the mitogen-activated protein kinase/extracellular signal-regulated kinase pathway by conventional, novel, and atypical protein kinase C isotypes. Mol. Cell. Biol..

[12] Zhou X, Quann E, Gallicano GI (2003). Differentiation of nonbeating embryonic stem cells into beating cardiomyocytes is dependent on downregulation of PKCβ and ζ in concert with upregulation of PKCε. Dev. Biol..

[13] Sachinidis A (2006). Identification of small signalling molecules promoting cardiac-specific differentiation of mouse embryonic stem cells. Cell. Physiol. Biochem..

[14] Feng X (2012). Protein kinase C mediated extraembryonic endoderm differentiation of human embryonic stem cells. Stem Cells.

[15] Mummery C (2003). Differentiation of human embryonic stem cells to cardiomyocytes: role of coculture with visceral endoderm-like cells. Circulation.

[16] Radaszkiewicz KA (2017). The acceleration of cardiomyogenesis in embryonic stem cells in vitro by serum depletion does not increase the number of developed cardiomyocytes. PLoS ONE.

[17] Veselá I, Kotasová H, Jankovská S, Procházková J, Pacherník J (2010). Leukaemia inhibitory factor inhibits cardiomyogenesis of mouse embryonic stem cells via STAT3 activation. Folia Biol. (Praha).

[18] Hsiao EC (2008). Marking embryonic stem cells with a 2A self-cleaving peptide: a NKX2-5 emerald GFP BAC reporter. PLoS ONE.

[19] Veeman MT, Slusarski DC, Kaykas A, Louie SH, Moon RT (2003). Zebrafish prickle, a modulator of noncanonical Wnt/Fz signaling, regulates gastrulation movements. Curr. Biol..

[20] Paclíková P, Bernatík O, Radaszkiewicz TW, Bryja V (2017). The N-terminal part of the dishevelled DEP domain is required for Wnt/β-catenin signaling in mammalian cells. Mol. Cell. Biol..

[21] Kučera J (2017). Hypoxia downregulates MAPK/ERK but Not STAT3 signaling in ROS-dependent and HIF-1-independent manners in mouse embryonic stem cells. Oxid. Med. Cell. Longev..

[22] Bisping E (2012). Transcription factor GATA4 is activated but not required for insulin-like growth factor 1 (IGF1)-induced cardiac hypertrophy. J. Biol. Chem..

[23] Li T (2019). The status of MAPK cascades contributes to the induction and activation of Gata4 and Nkx2.5 during the stepwise process of cardiac differentiation. Cell. Signal..

[24] Jeyarajah MJ, Jaju Bhattad G, Kops BF, Renaud SJ (2019). Syndecan-4 regulates extravillous trophoblast migration by coordinating protein kinase C activation. Sci. Rep..

[25] Virant D (2018). A peptide tag-specific nanobody enables high-quality labeling for dSTORM imaging. Nat. Commun..

[26] Radaszkiewicz T, Bryja V, Bryja V (2020). Protease associated domain of RNF43 is not necessary for the suppression of Wnt/β-catenin signaling in human cells. Cell Commun. Signal..

[27] Kotoku T (2016). CIBZ regulates mesodermal and cardiac differentiation of by suppressing T and Mesp1 expression in mouse embryonic stem cells. Sci. Rep..

[28] Millius A, Weiner OD (2010). Manipulation of neutrophil-like HL-60 cells for the study of directed cell migration. Methods Mol. Biol..

[29] Souček K (2006). Transforming growth factor-β1 inhibits all-trans retinoic acid-induced apoptosis. Leuk. Res..

[30] Pavelkova M, Kubala L (2004). Luminol-, isoluminol- and lucigenin-enhanced chemiluminescence of rat blood phagocytes stimulated with different activators. Luminescence.

[31] England J, Loughna S (2013). Heavy and light roles: myosin in the morphogenesis of the heart. Cell. Mol. Life Sci..

[32] Burridge PW (2014). Chemically defined generation of human cardiomyocytes. Nat. Methods.

[33] Elliott DA (2011). NKX2-5eGFP/w hESCs for isolation of human cardiac progenitors and cardiomyocytes. Nat. Methods.

[34] Moses KA, Demayo F, Braun RM, Reecy JL, Schwartz RJ (2001). Embryonic expression of an Nkx2-5/Cre gene using ROSA26 reporter mice. Genesis.

[35] Lin Q, Schwarz J, Bucana C, Olson EN (1997). Control of mouse cardiac morphogenesis and myogenesis by transcription factor MEF2C. Science.

[36] Bruneau BG (2002). Transcriptional regulation of vertebrate cardiac morphogenesis. Circ. Res..

[37] Ramírez-Bergeron DL (2004). Hypoxia affects mesoderm and enhances hemangioblast specification during early development. Development.

[38] Kitajima S, Takagi A, Inoue T, Saga Y (2000). MesP1 and MesP2 are essential for the development of cardiac mesoderm. Development.

[39] Liu Y (2016). Mesp1 marked cardiac progenitor cells repair infarcted mouse hearts. Sci. Rep..

[40] Devine WP, Wythe JD, George M, Koshiba-Takeuchi K, Bruneau BG (2014). Early patterning and specification of cardiac progenitors in gastrulating mesoderm. Elife.

[41] Keranen LM, Dutil EM, Newton AC (1995). Protein kinase C is regulated in vivo by three functionally distinct phosphorylations. Curr. Biol..

[42] Shaul YD, Seger R (2007). The MEK/ERK cascade: from signaling specificity to diverse functions. Biochim. Biophys. Acta Mol. Cell Res..

[43] Gregg J, Fraizer G (2011). Transcriptional regulation of EGR1 by EGF and the ERK signaling pathway in prostate cancer cells. Genes Cancer.

[44] Katanasaka Y, Suzuki H, Sunagawa Y, Hasegawa K, Morimoto T (2016). Regulation of cardiac transcription factor GATA4 by post-translational modification in cardiomyocyte hypertrophy and heart failure. Int. Heart J..

[45] Lim PS, Sutton CR, Rao S (2015). Protein kinase C in the immune system: from signalling to chromatin regulation. Immunology.

[46] Rybin VO, Steinberg SF (1994). Protein kinase C isoform expression and regulation in the developing rat heart. Circ. Res..

[47] Hug H, Sarre TF (1993). Protein kinase C isoenzymes: divergence in signal transduction?. Biochem. J..

[48] Basu A, Pal D (2010). Two faces of protein kinase Cδ: the contrasting roles of PKCδ in cell survival and cell death. Sci. World J..

[49] Morrison DK (2012). MAP kinase pathways. Cold Spring Harb. Perspect. Biol..

[50] Gallicano GI, Mobley S, Shookhof JM, Foshay K, Park M (2010). PKG and PKC are down-regulated during cardiomyocyte differentiation from embryonic stem cells: manipulation of these pathways enhances cardiomyocyte production. Stem Cells Int..

[51] Xu FY, Fandrich RR, Nemer M, Kardami E, Hatch GM (1999). The subcellular distribution of protein kinase Cα, -ε, and -ζ isoforms during cardiac cell differentiation. Arch. Biochem. Biophys..

[52] Ventura C, Zinellu E, Maninchedda E, Fadda M, Maioli M (2003). Protein kinase C signaling transduces endorphin-primed. Circ. Res..

[53] Lin H-Y, Lee D-C, Wang H-D, Chi Y-H, Chiu I-M (2015). Activation of FGF1B promoter and FGF1 are involved in cardiogenesis through the signaling of PKC, but not MAPK. Stem Cells Dev..

[54] Rose BA, Force T, Wang Y (2010). Mitogen-activated protein kinase signaling in the heart: angels versus demons in a heart-breaking tale. Physiol. Rev..

[55] Herbert JM, Augereau JM, Gleye J, Maffrand JP (1990). Chelerythrine is a potent and specific inhibitor of protein kinase C. Biochem. Biophys. Res. Commun..

[56] Wu-Zhang AX, Newton AC (2013). Protein kinase C pharmacology: refining the toolbox. Biochem. J..

[57] Kuwabara WMT (2015). NADPH oxidase-dependent production of reactive oxygen species induces endoplasmatic reticulum stress in neutrophil-like HL60 cells. PLoS ONE.

[58] Vrba J, Dvořák Z, Ulrichová J, Modrianský M (2008). Conventional protein kinase C isoenzymes undergo dephosphorylation in neutrophil-like HL-60 cells treated by chelerythrine or sanguinarine. Cell Biol. Toxicol..

[59] Dirkx E, da Costa Martins PA, De Windt LJ (2013). Regulation of fetal gene expression in heart failure. Biochim. Biophys. Acta Mol. Basis Dis..

[60] Ryan K, Chin AJ (2003). T-box genes and cardiac development. Birth Defects Res. Part C Embryo Today Rev..

[61] Bondue A (2008). Mesp1 acts as a master regulator of multipotent cardiovascular progenitor specification. Cell Stem Cell.

[62] Lindsley RC (2008). Mesp1 coordinately regulates cardiovascular fate restriction and epithelial-mesenchymal transition in differentiating ESCs. Cell Stem Cell.

[63] Oliva JL (2005). PKC isozymes and diacylglycerol-regulated proteins as effectors of growth factor receptors. Growth Factors.

[64] Nath S, Devi GR (2016). Three-dimensional culture systems in cancer research: focus on tumor spheroid model. Pharmacol. Ther..

[65] Van Winkle AP, Gates ID, Kallos MS (2012). Mass transfer limitations in embryoid bodies during human embryonic stem cell differentiation. Cells Tissues Organs.

[66] Gessert S, Kühl M (2010). The multiple phases and faces of wnt signaling during cardiac differentiation and development. Circ. Res..

[67] Červenka I (2011). Mitogen-activated protein kinases promote WNT/beta-catenin signaling via phosphorylation of LRP6. Mol. Cell. Biol..

