# Supplementary material for: 12-O-Tetradecanoylphorbol-13-acetate increases cardiomyogenesis through PKC/ERK signaling
Source: Sci Rep. 2020 Sep 28;10:15922. doi: 10.1038/s41598-020-73074-4 (PMC7522207; doi:10.1038/s41598-020-73074-4)
Supplement: Supplementary file 1 — Supplementary Figures. [file 41598_2020_73074_MOESM1_ESM.pdf]

12-O-Tetradecanoylphorbol-13-acetate increases cardiomyogenesis through PKC/ERK signaling

Katarzyna Anna Radaszkiewicz[1], Deborah Beckerová[1], Lucie Woloszczuková[1], Tomasz Witold Radaszkiewicz[1], Petra Lesáková[1], Olga Vondálová Blanářová[1], Lukáš Kubala[1,2], Petr Humpolíček[3], Jiří Pachernik[1*]

[1] Department of Experimental Biology, Faculty of Science, Masaryk University, Brno, Czech Republic,

[2] Department of Free Radical Pathophysiology, Institute of Biophysics, Academy of Sciences of the Czech Republic, Brno, Czech Republic,

[3] Centre of Polymer Systems and Faculty of Technology, Tomas Bata University in Zlin, 760 01 Zlin, Czech Republic

[*]Corresponding author: jipa@sci.muni.cz

Supplementary Fig. 1

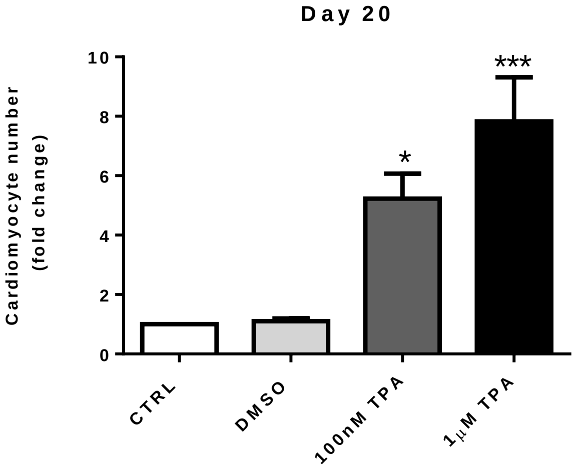

Supplementary Fig.1. The effect of TPA treatment on the number of derived cardiomyocytes – indirect quantification. The number of cardiomyocytes was determined in HG8 cells treated with 0.1% DMSO, 100 nM TPA and 1 µM TPA between Day 6 and Day 8 of differentiation. The selection of cardiomyocytes began on Day 14 and measurements were performed on Day 20 of differentiation. Data are presented as mean ± SEM, n≥3. Statistical significance was determined by ANOVA with post hoc Bonferroni's Multiple Comparison test; * $P < 0.05$; ** $P < 0.01$; *** $P < 0.001$.

Supplementary Fig. 2

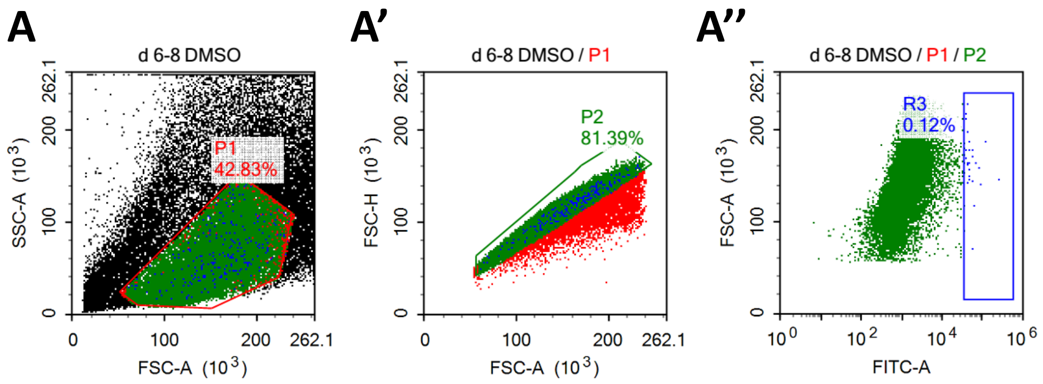

Supplementary Fig.2. The effect of TPA treatment on the number of derived cardiomyocytes – direct quantification. R1 cells were treated with 1 µM of TPA on Days 6-8 (d 6-8) Days 10-12 (d 10-12) of differentiation. Flow cytometric quantification of myosine heavy chains (MHCs) was performed on Day 20. Gating strategy: Exclusion of debris on the forward and side scatter (FSC-A x SSC-A), gate P1 (A), exclusion of doublets on the forward scatter (FSC-A x FSC-H), gate P2 (A’). Gating of MHC-positive population (FSC-A x FITC-A), gate R3 (A’’).

Supplementary Fig. 3

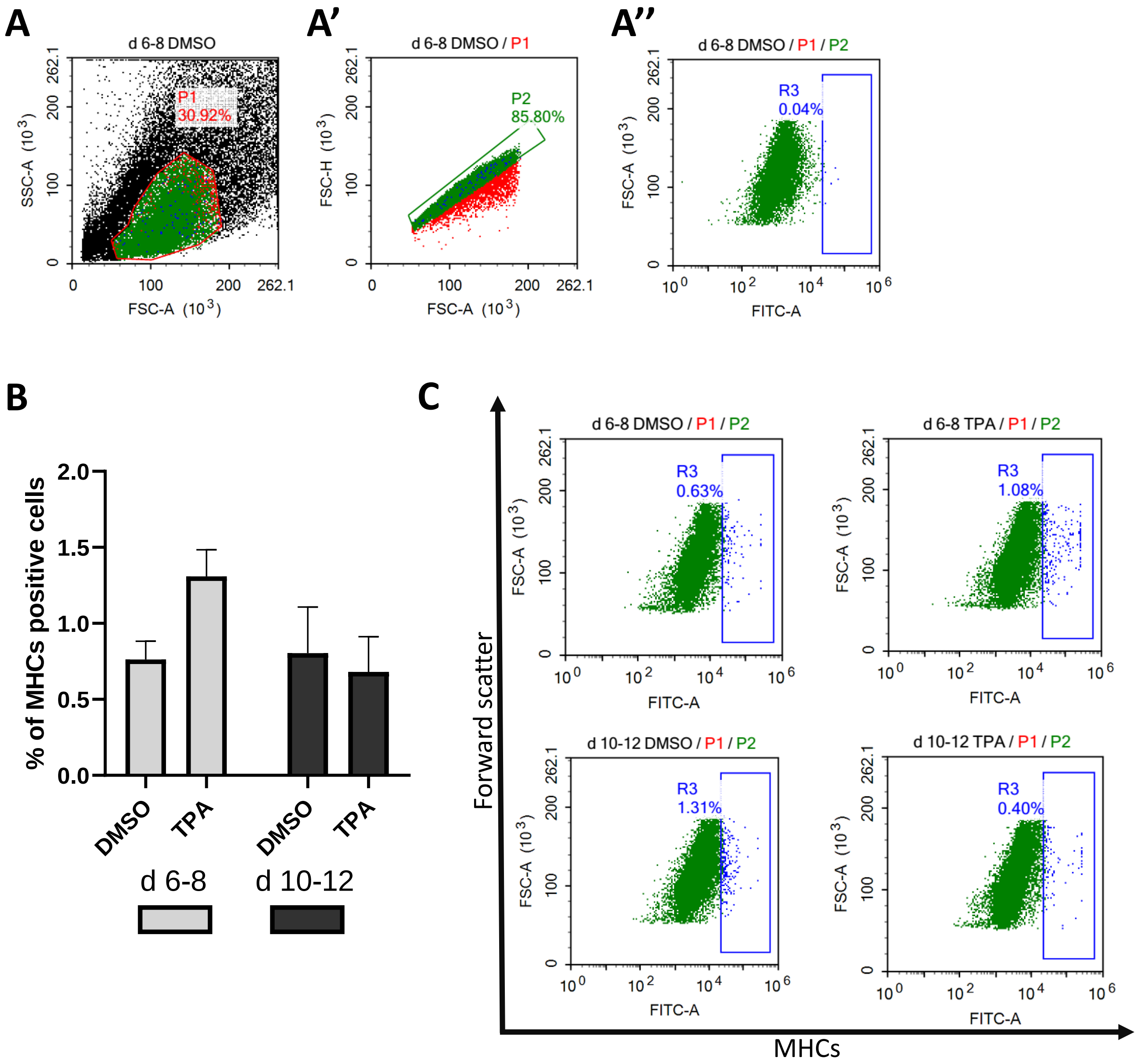

Supplementary Fig.3. The effect of TPA treatment on the number of derived cardiomyocytes – direct quantification. D3 cells were treated with 1 µM of TPA on Days 6-8 (d 6-8) Days 10-12 (d 10-12) of differentiation. Flow cytometric quantification of myosine heavy chains (MHCs) was performed on Day 20. Gating strategy: Exclusion of debris on the forward and side scatter (FSC-A x SSC-A), gate P1 (A), exclusion of doublets on the forward scatter (FSC-A x FSC-H), gate P2 (A'). Gating of MHC-positive population (FSC-A x FITC-A) based on negative control, gate R3 (A''). Percentage of MHC-positive cells (B). Representative plots; gate R3 shows the percentage of MHC-positive cells (C). Data are presented as mean ± SEM, n≥3. Statistical significance was determined by t-test; * $P < 0.05$; ** $P < 0.01$; *** $P < 0.001$.

Supplementary Fig. 4

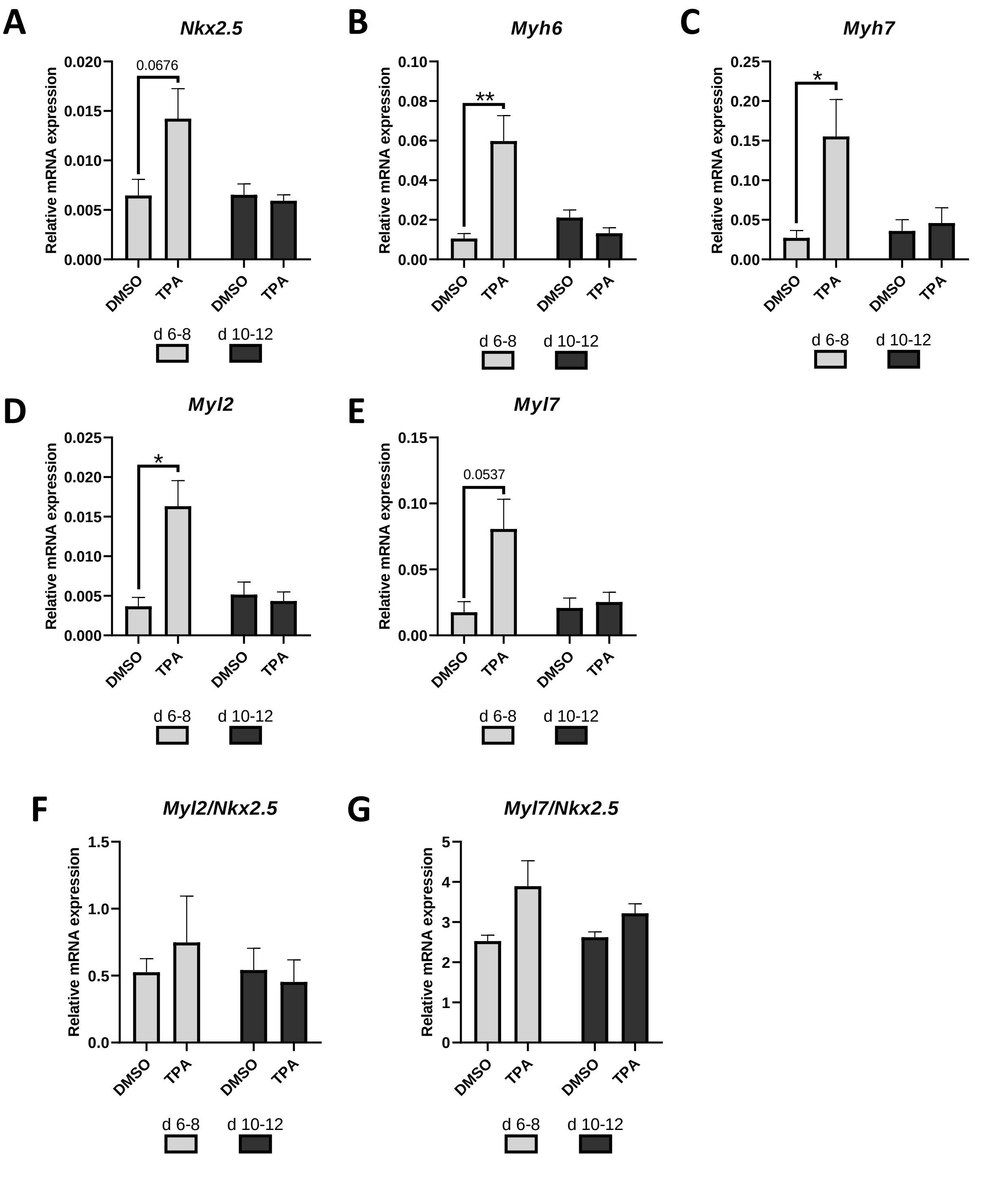

Supplementary Fig.4. The effect of TPA treatment on the expression of cardiomyocyte specific transcripts. Relative mRNA levels in D3 cells differentiated for 20 days and treated by 0.1% DMSO and 1 μM TPA between Day 6 and Day 8 of differentiation, determined by qRT-PCR normalised to the mean expression of *Hprt* and *Rpl13a* genes (A-E) or normalised to the *Nkx2.5* gene (F, G). Data are presented as mean ± SEM, n≥3. Statistical significance was determined by ANOVA with post hoc Bonferroni's Multiple Comparison test; * $P < 0.05$; ** $P < 0.01$; *** $P < 0.001$.

Supplementary Fig. 5

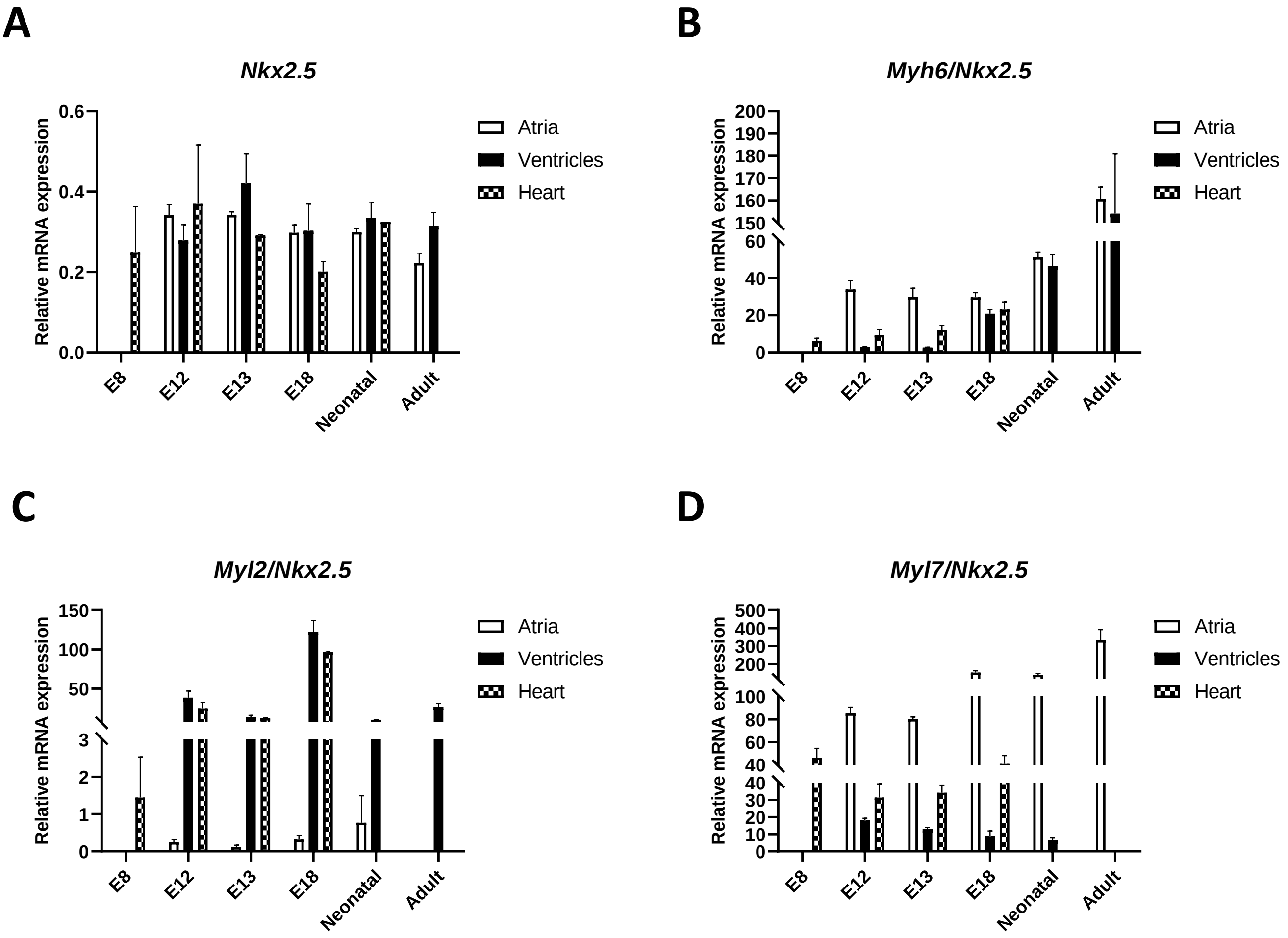

Supplementary Fig.5. Cardiac gene expression during mouse heart development. Relative mRNA levels in atria, ventricles, and whole heart in different stages of heart development (embryonic day 8,12,13,18, neonatal and adult heart), determined by qRT-PCR normalised to the mean expression of *Hprt* and *Rpl13a* genes (A) or normalised to the *Nkx2.5* gene (C-D). Data are presented as mean ± SEM, n≥3.

Supplementary Fig. 6

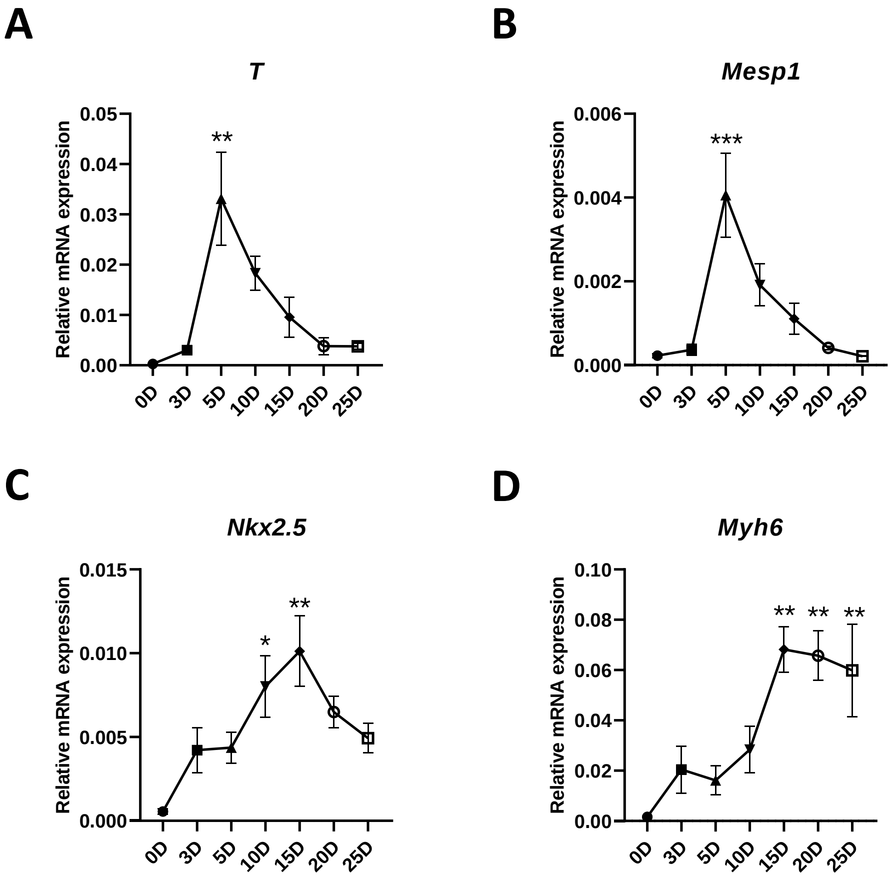

Supplementary Fig.6. Gene expression during cardiac differentiation.
Relative mRNA levels in R1 cells differentiated for 25 days determined by qRT-PCR normalised to the mean expression of *Hprt* and *Rpl13a* genes. Marker of mesoderm (A), cardiac mesoderm (B), committed cardiac progenitors (C), and cardiomyocytes (D). Data are presented as mean ± SEM, n≥3. Statistical significance was determined by ANOVA with post hoc Bonferroni's Multiple Comparison test; * $P < 0.05$; ** $P < 0.01$; *** $P < 0.001$.

Supplementary Fig. 7

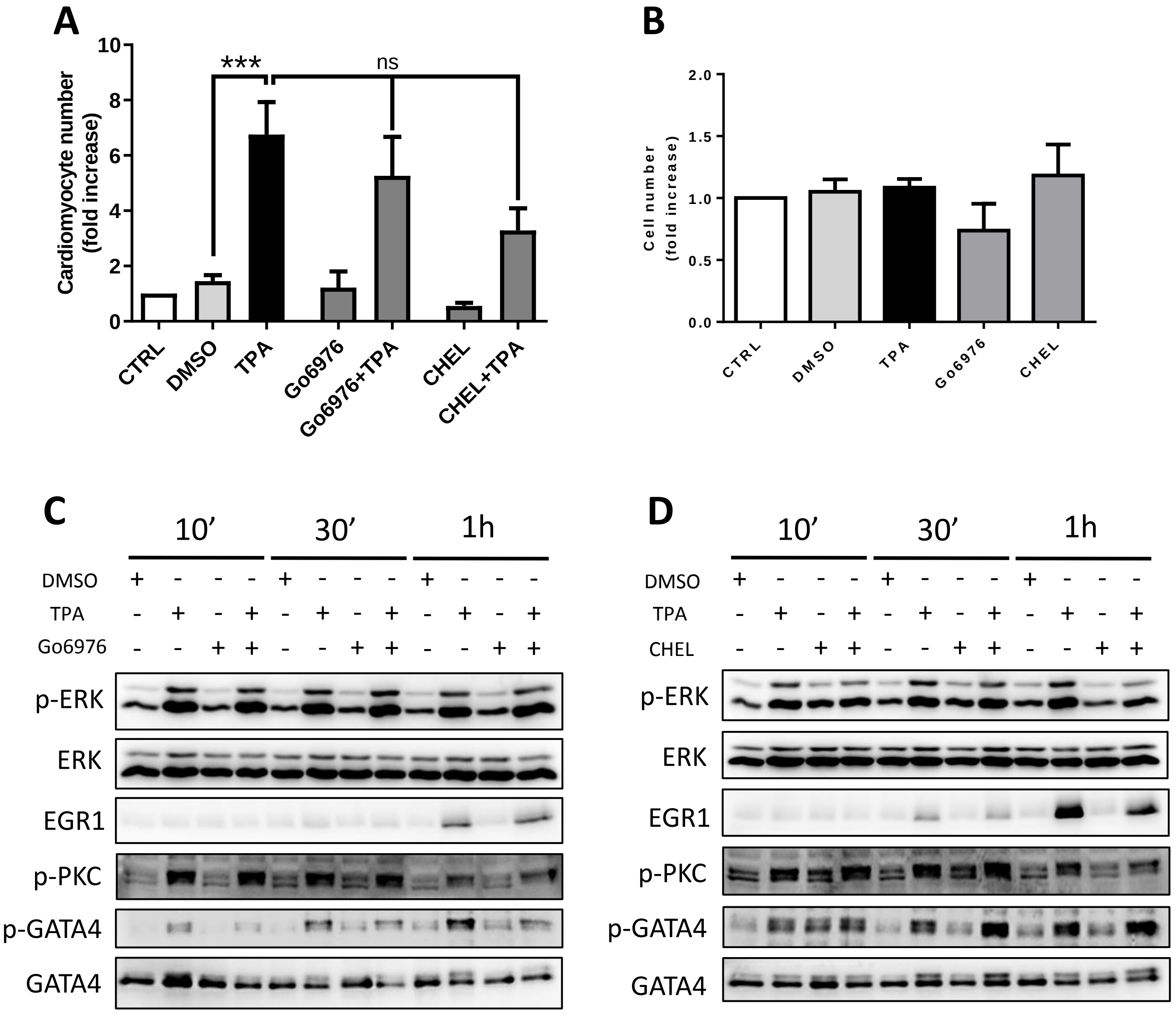

Supplementary Fig.7. The role of PKC and ERK signalling pathways in TPA-induced cardiomyogenesis. The number of cardiomyocytes was determined after treatments between Day 6 and Day 8 of HG8 cell with the following inhibitors: 1 µM Go6976 and 5 µM chelerythrine chloride (CHEL) in combination with 1 µM TPA and 0.1% DMSO as solvent control. Selection of cardiomyocytes began on Day 14 and measurements were performed on Day 20 of differentiation (A). Fold of change in cell number for the non-selected population on Day 14 of differentiation after treatments corresponding to “A” (B). Western blot analysis of the phosphorylation status of PKC, ERK, and GATA4, and the level of EGR1 after treatment of 6-day-old embryoid bodies (R1 cell line) with 0.1% DMSO or 1 µM TPA and the aforementioned inhibitors in the indicated time intervals (C, D). Data are presented as mean ± SEM, n≥3. Statistical significance was determined by ANOVA with post hoc Bonferroni's Multiple Comparison test; * $P < 0.05$; ** $P < 0.01$; *** $P < 0.001$.

Supplementary Fig. 8

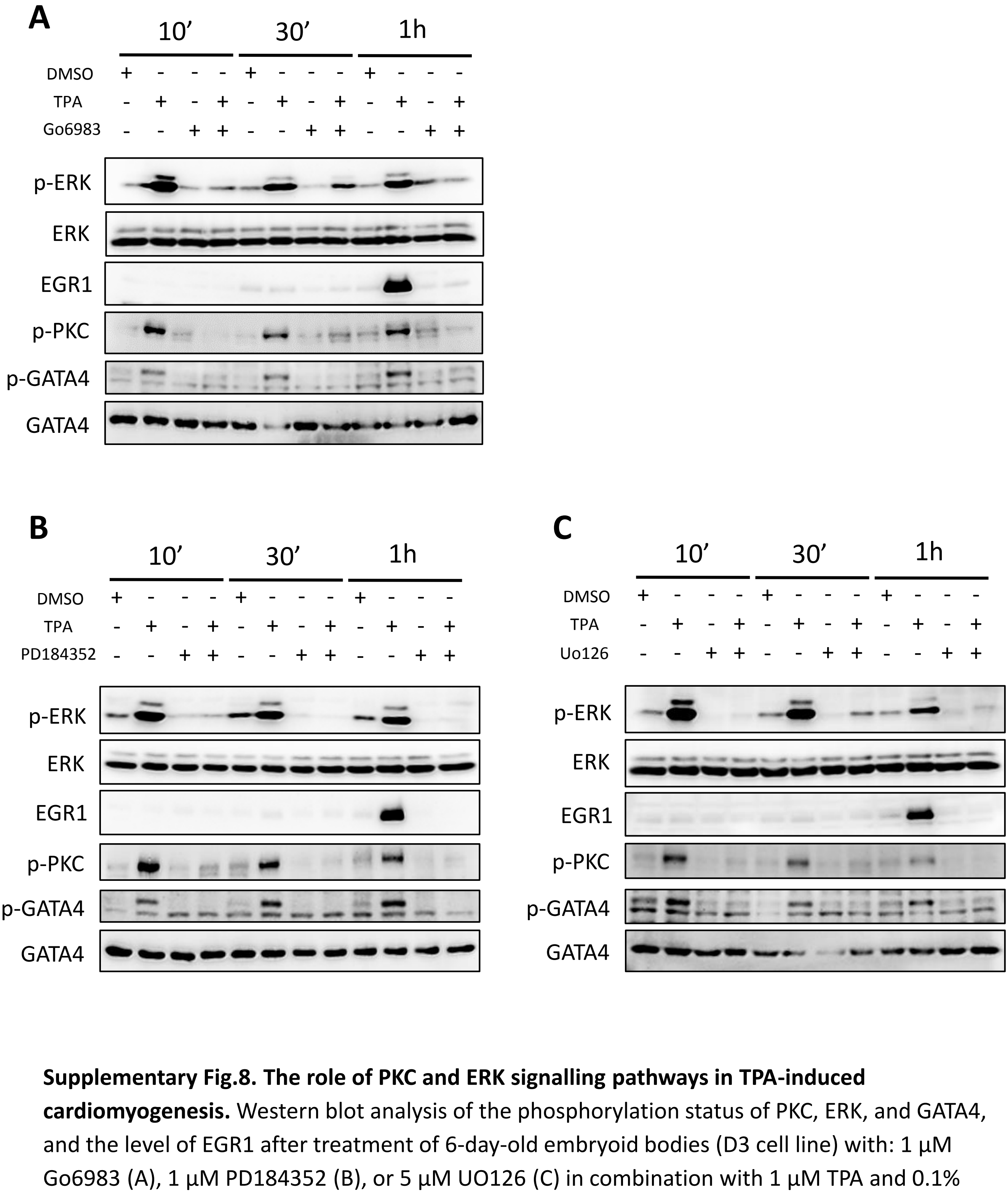

Supplementary Fig.8. The role of PKC and ERK signalling pathways in TPA-induced cardiomyogenesis. Western blot analysis of the phosphorylation status of PKC, ERK, and GATA4, and the level of EGR1 after treatment of 6-day-old embryoid bodies (D3 cell line) with: 1 µM Go6983 (A), 1 µM PD184352 (B), or 5 µM UO126 (C) in combination with 1 µM TPA and 0.1% DMSO as solvent control in the indicated time intervals, n≥3.

Supplementary Fig. 9

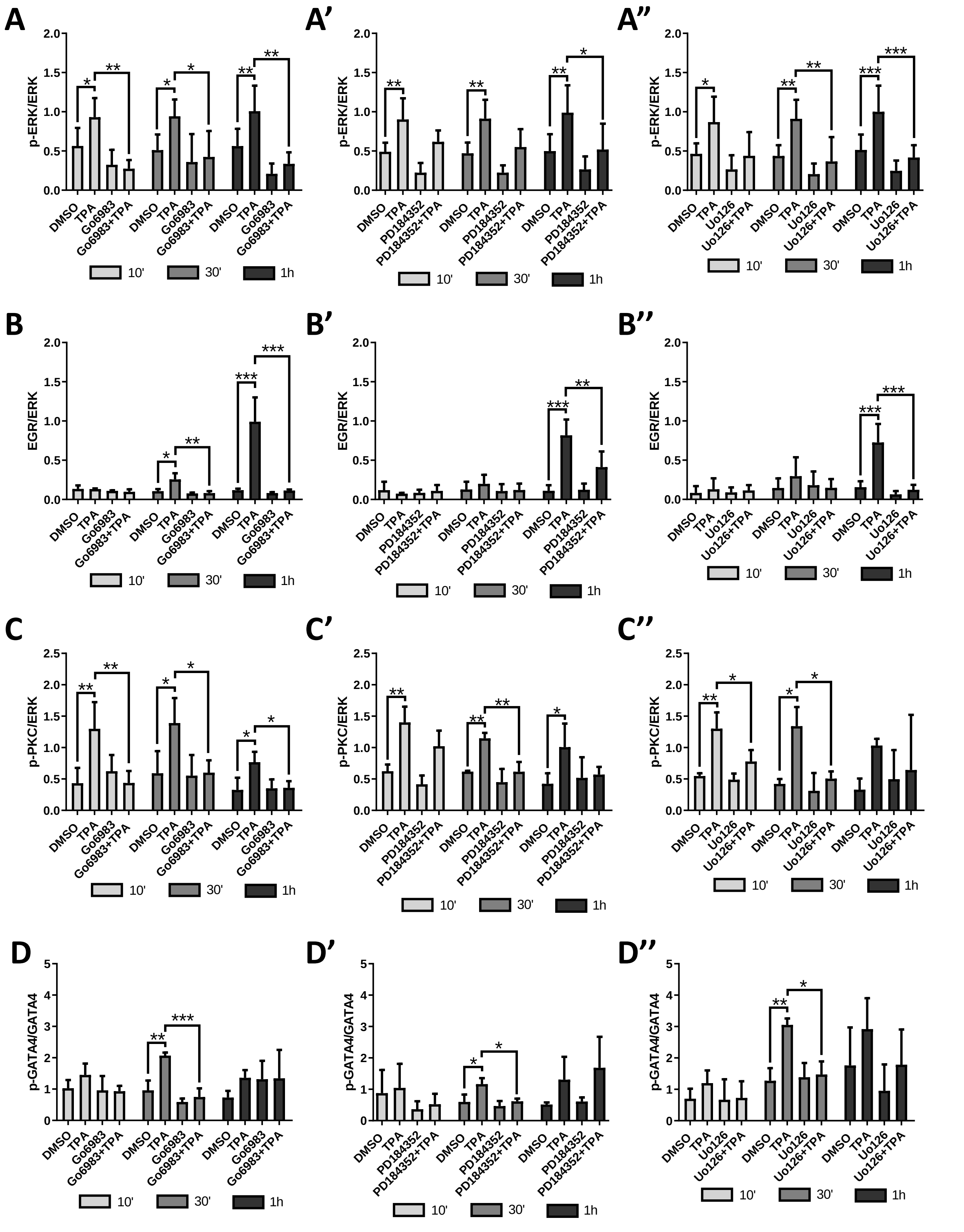

Supplementary Fig.9. The role of PKC and ERK signalling pathways in TPA-induced cardiomyogenesis. Relative quantification of EGR1, p-PKC, p-GATA4 Western blot signal determined by densitometric analysis. R1 cell line-derived 6-day-old embryoid bodies were treated with: 1 µM Go6983 (A-C), 1 µM PD184352 (A'-C'), or 5 µM UO126 (A''-C'') in combination with 1 µM TPA and 0.1% DMSO as solvent control in the indicated time intervals. Data are presented as mean ± SEM, n≥3. Statistical significance was determined by ANOVA with post hoc Bonferroni's Multiple Comparison test; * $P < 0.05$; ** $P < 0.01$; *** $P < 0.001$.

Supplementary Fig. 10

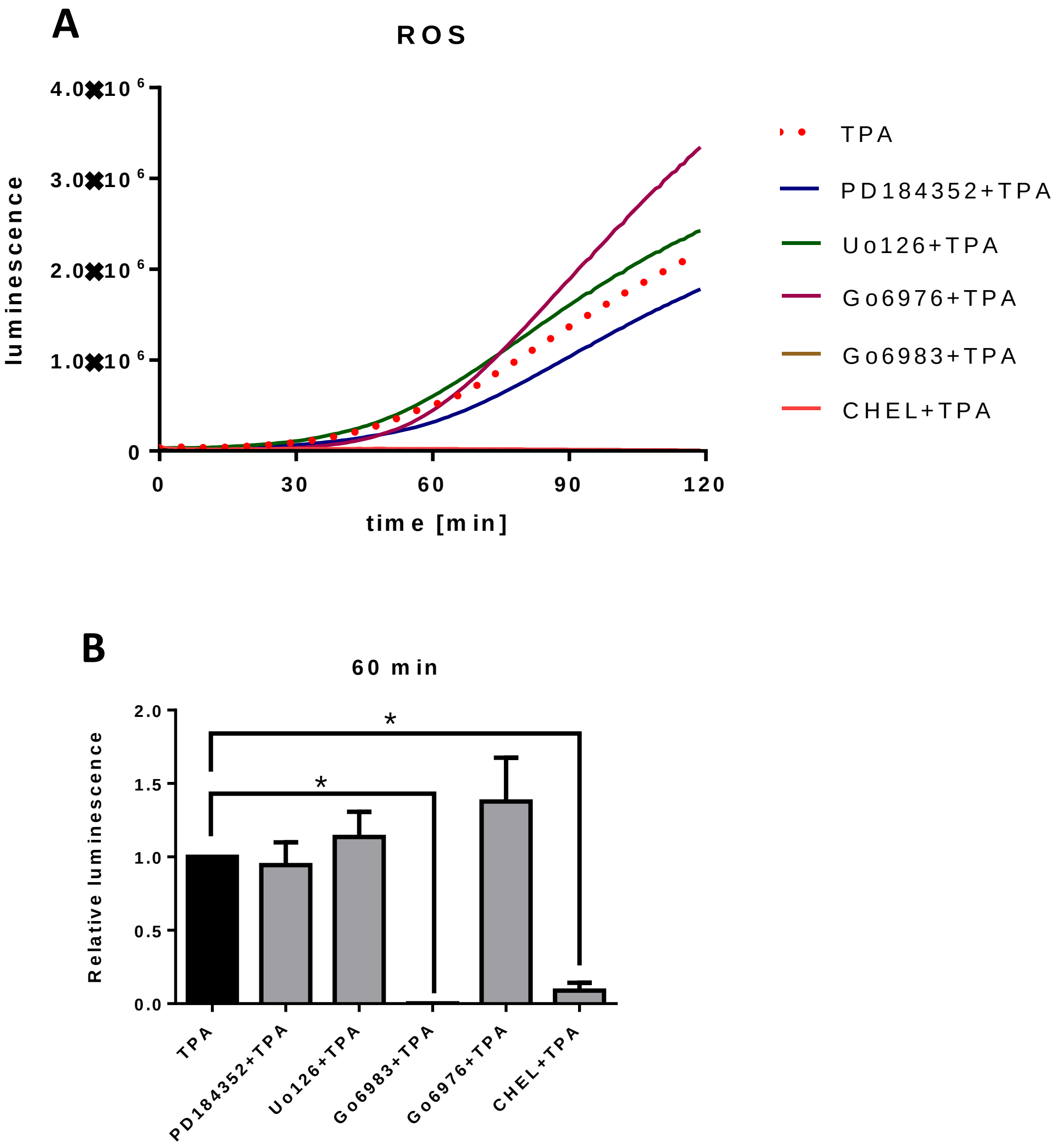

Supplementary Fig.10. PKC and ERK inhibitor validation by ROS production assay.
The kinetic profiles of ROS production from one representative experiment. Differentiated HL60 cells were pre-incubated with 1 µM PD184352, 5 µM UO126, 1 µM Go6983, 1 µM Go6976 or 5 µM chelerythrine chloride (CHEL) for 10 minutes and consequently treated with 1 µM TPA (A). Data acquired 60 min after cell activation by TPA with the aforementioned inhibitors (B). Data are normalised to the TPA-only condition. Data are presented as mean ± SEM, n≥3. Statistical significance was determined by ANOVA with post hoc Bonferroni's Multiple Comparison test; * P < 0.05; ** P < 0.01; *** P < 0.001.

Supplementary Fig. 11

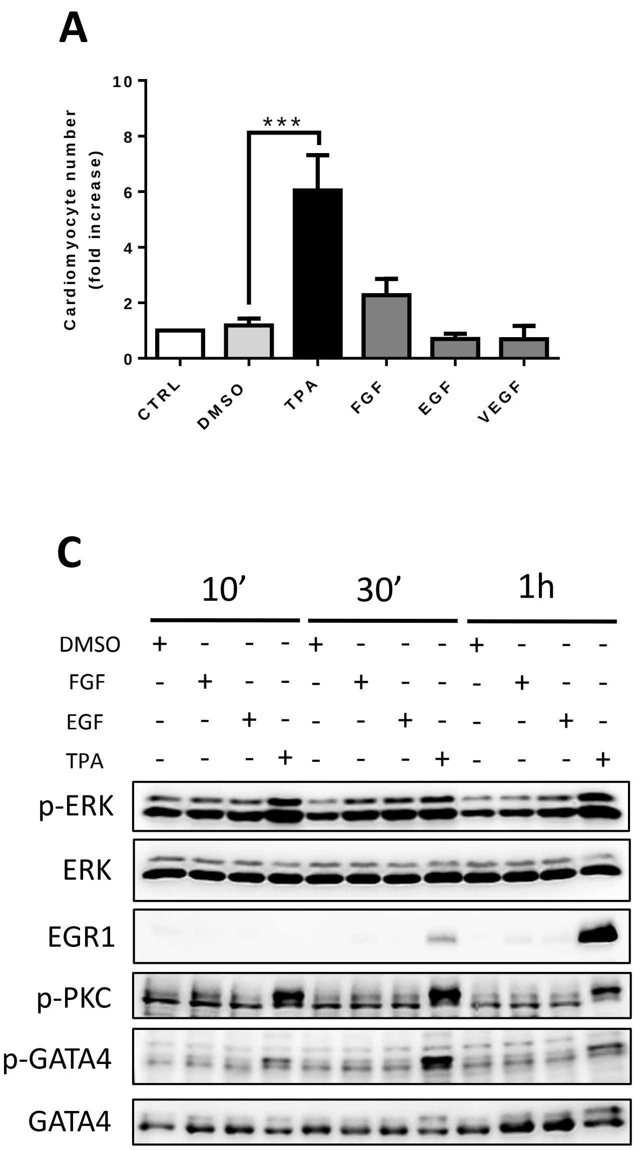

Supplementary Fig.11. The role of selected ERK signalling pathway induced growth factors in TPA-induced cardiomyogenesis

The number of cardiomyocytes was determined after treatments between Day 6 and Day 8 of HG8 cells with the following growth factors: 10 ng/ml FGF (fibroblast growth factor), 10 ng/ml EGF (epidermal growth factor), 10 ng/ml VEGF (vascular endothelial growth factor) (A) or different concentrations of FBS (fetal calf serum) (B) in combination with 1 μM TPA and 0.1% DMSO as solvent control. Western blot analysis of the phosphorylation status of PKC, ERK, and GATA4, and the level of EGR1 after treatment of 6-day-old embryoid bodies (R1 cell line) with 0.1% DMSO or 1 μM TPA and the aforementioned growth factors in the indicated time intervals (C). Data are presented as mean ± SEM, n≥3. Statistical significance was determined by ANOVA with post hoc Bonferroni's Multiple Comparison test; * P < 0.05; ** P < 0.01; *** P < 0.001.

Supplementary Fig. 12

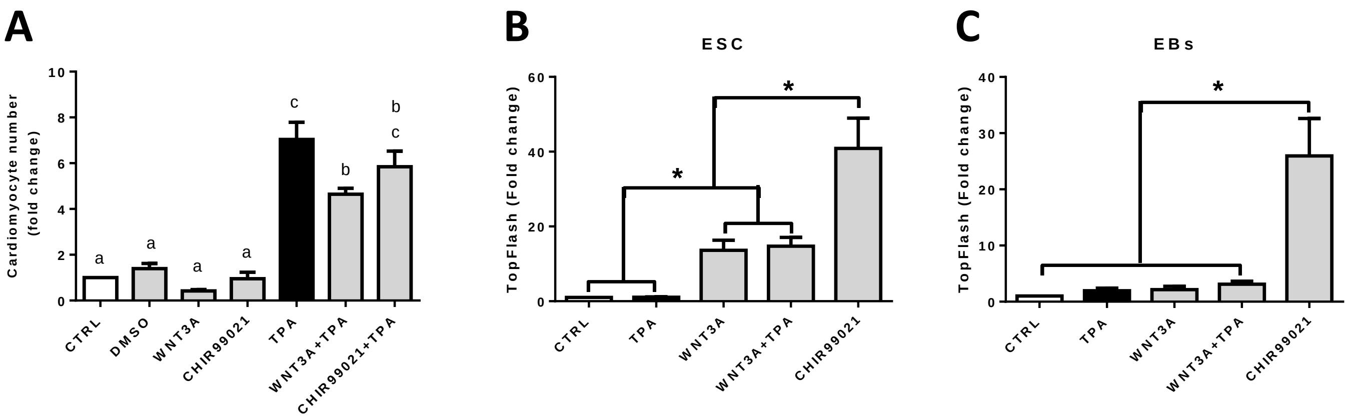

Supplementary Fig.12. The role of canonical WNT signalling in the TPA-mediated effect on cardiomyogenesis.

The number of cardiomyocytes was determined after treatment of HG8 cells with 0.1 % DMSO, 1 µM TPA, 100 ng/ml WNT3A, 2 µM CHIR99021 and a combination of TPA with WNT3A/CHIR99021 (A). The selection of cardiomyocytes began on Day 14 and measurements were performed on Day 20 of differentiation. Embryonic stem cells (B) and embryoid bodies (C) derived from TfR1 cells were treated with 1 µM TPA, conditional WNT3A medium, 2 µM CHIR99021, and a combination of TPA with WNT3A conditional medium. The activity of β-catenin dependent WNT signalling was measured by TOPflash reporter assay. Data are presented as mean± SEM, n≥3. Statistical significance was determined by ANOVA with post hoc Bonferroni's Multiple Comparison test. The groups marked differently by symbol letters or by “*“ are statistically significantly different from each other; $P < 0.05$.

Supplementary Fig. 13

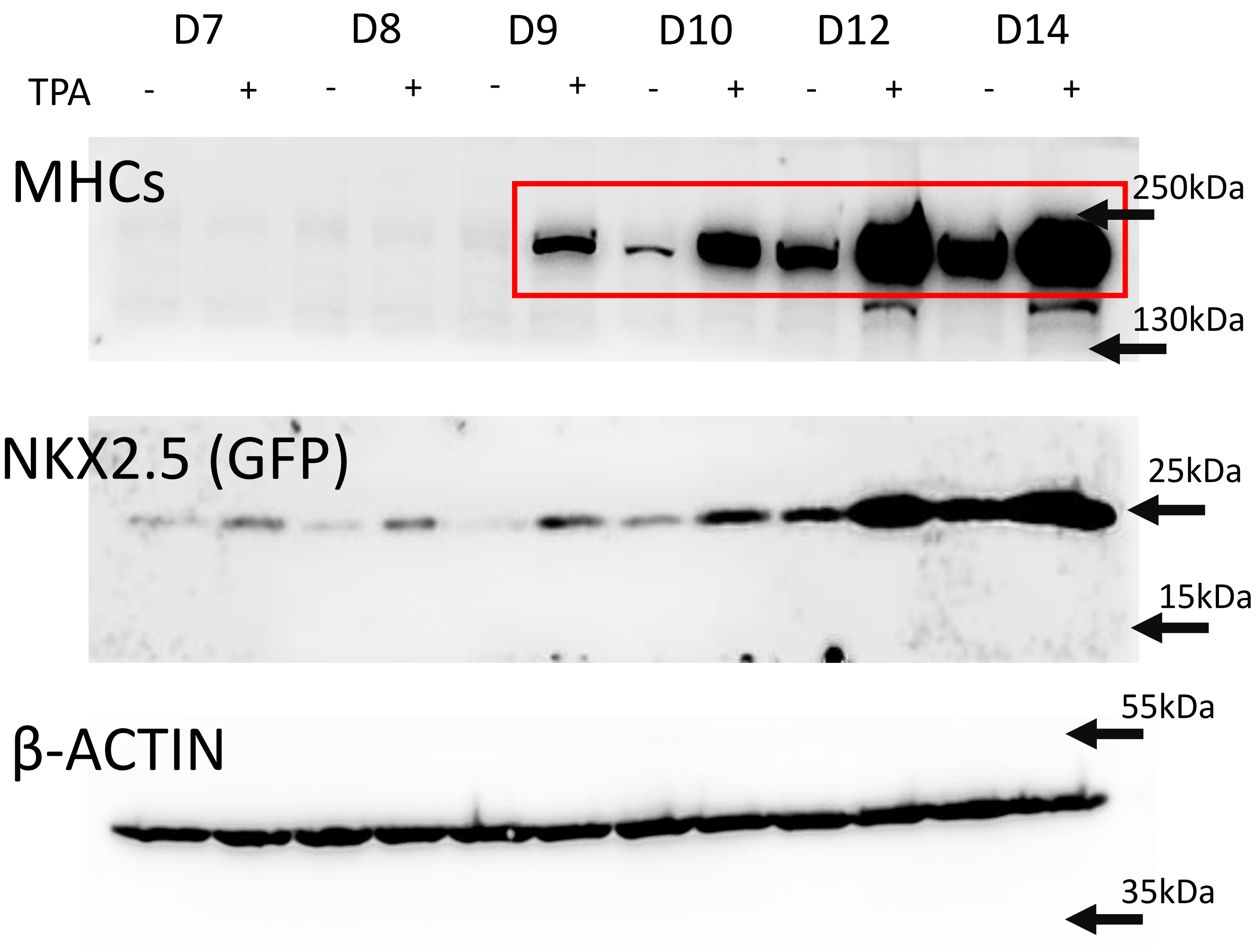

Supplementary Fig.13. Unprocessed Western blot membranes with molecular mass marker related to Fig. 5F. Bands surrounded by red frames are specific.

Supplementary Fig. 14

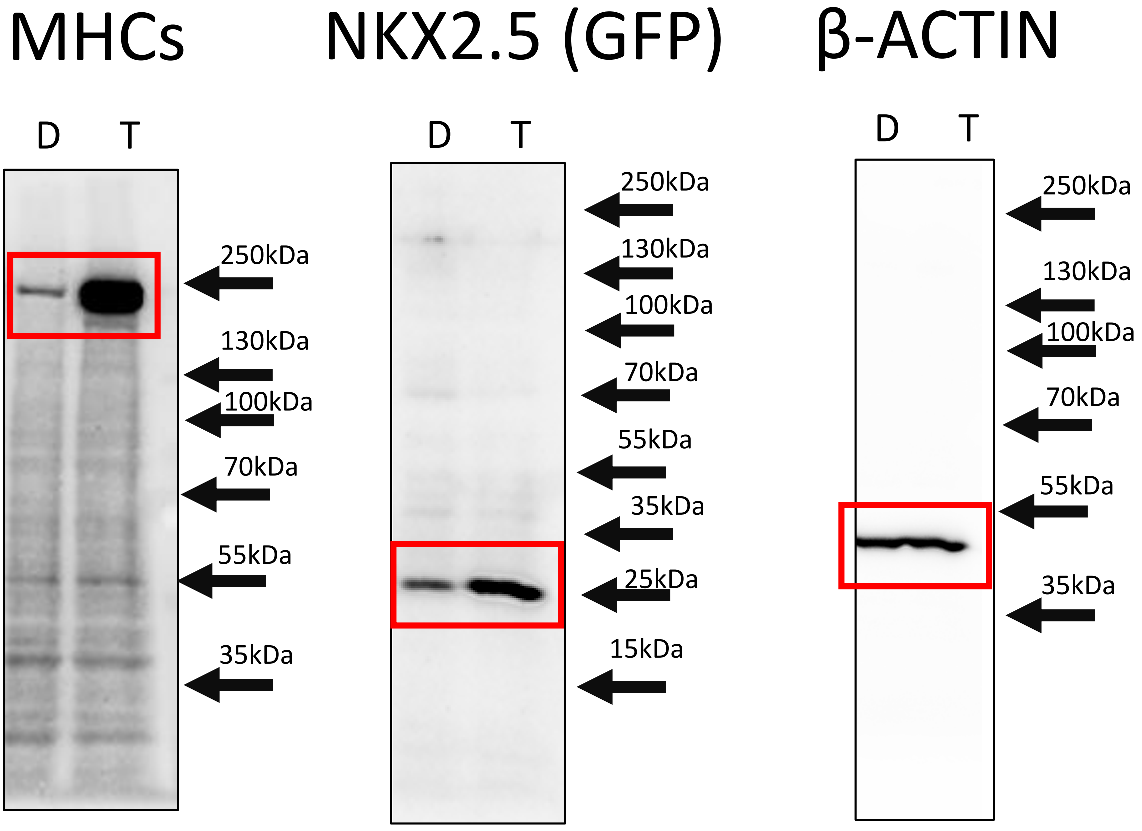

Supplementary Fig.14. Representative full-length Western blot membranes with molecular mass marker. Western blot analysis of myosine heavy chains (MHCs) and Nkx2.5 level (as level of GFP) at day 14 of NK4 cell differentiation. Cells were treated with 0.1% DMSO (marked as D) and 1 µM TPA (marked as T) between Day 6 and Day 8 of differentiation. Bands surrounded by red frames were taken as specific.

Supplementary Fig. 15

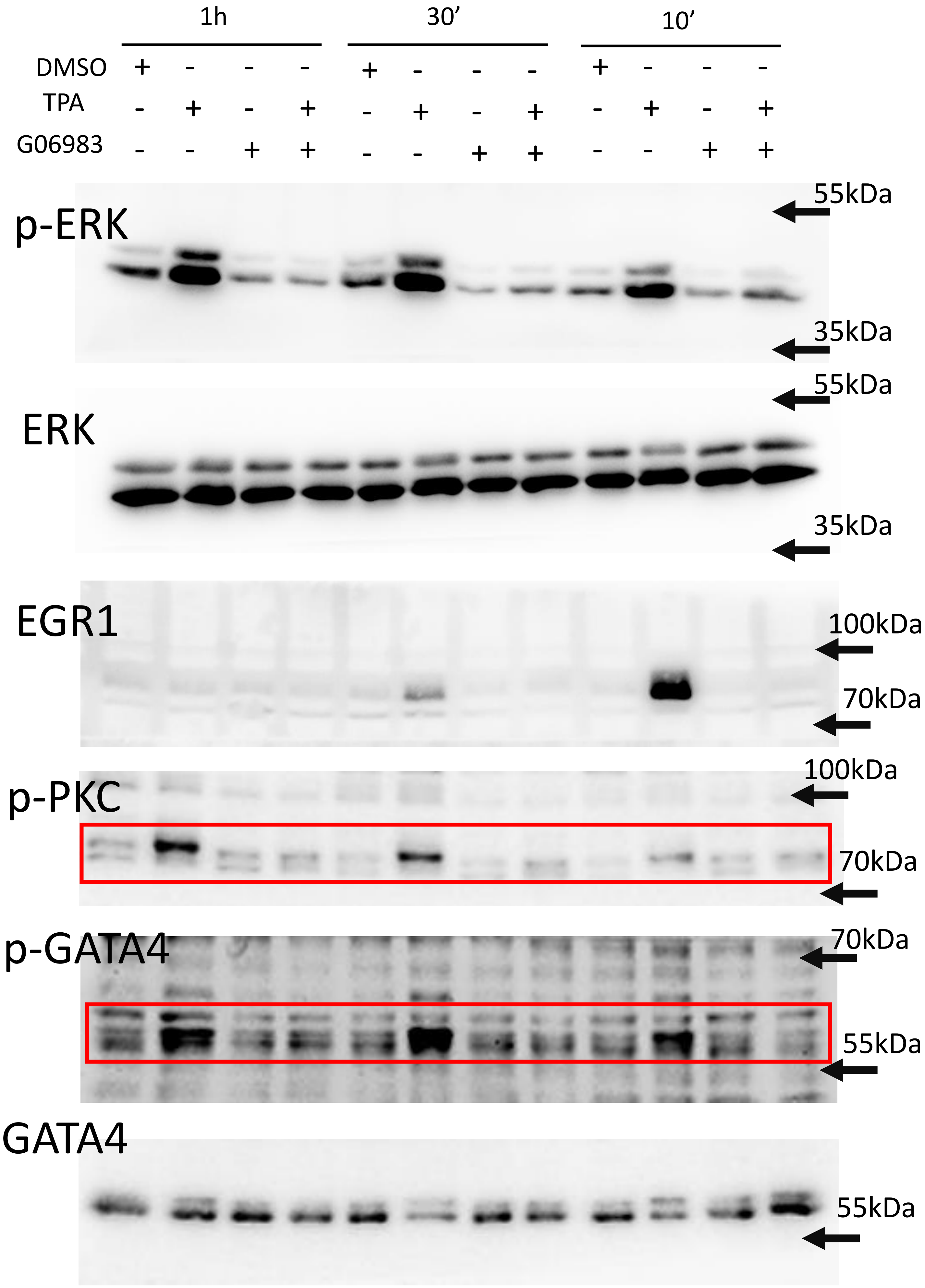

Supplementary Fig. 15

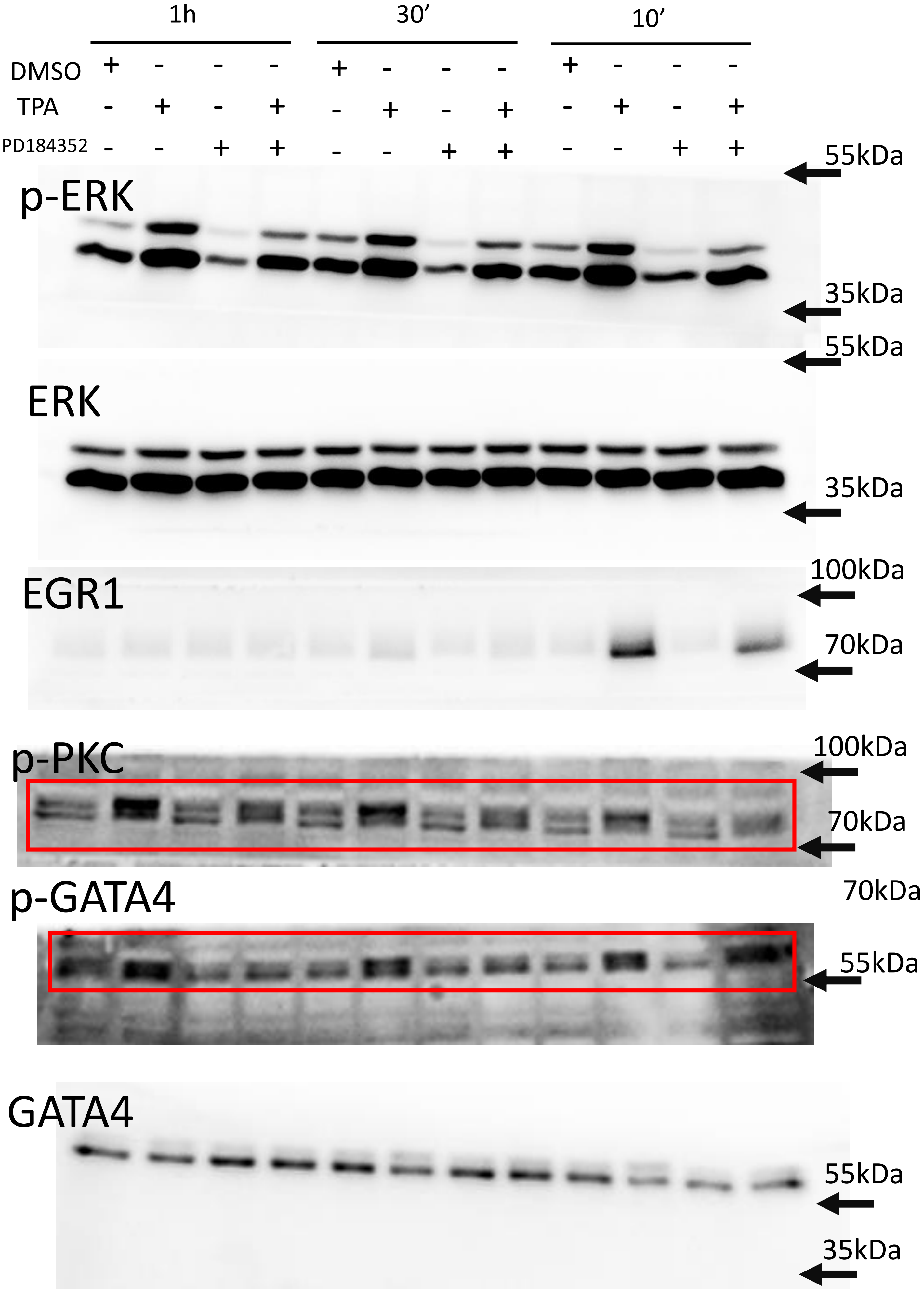

Supplementary Fig. 15

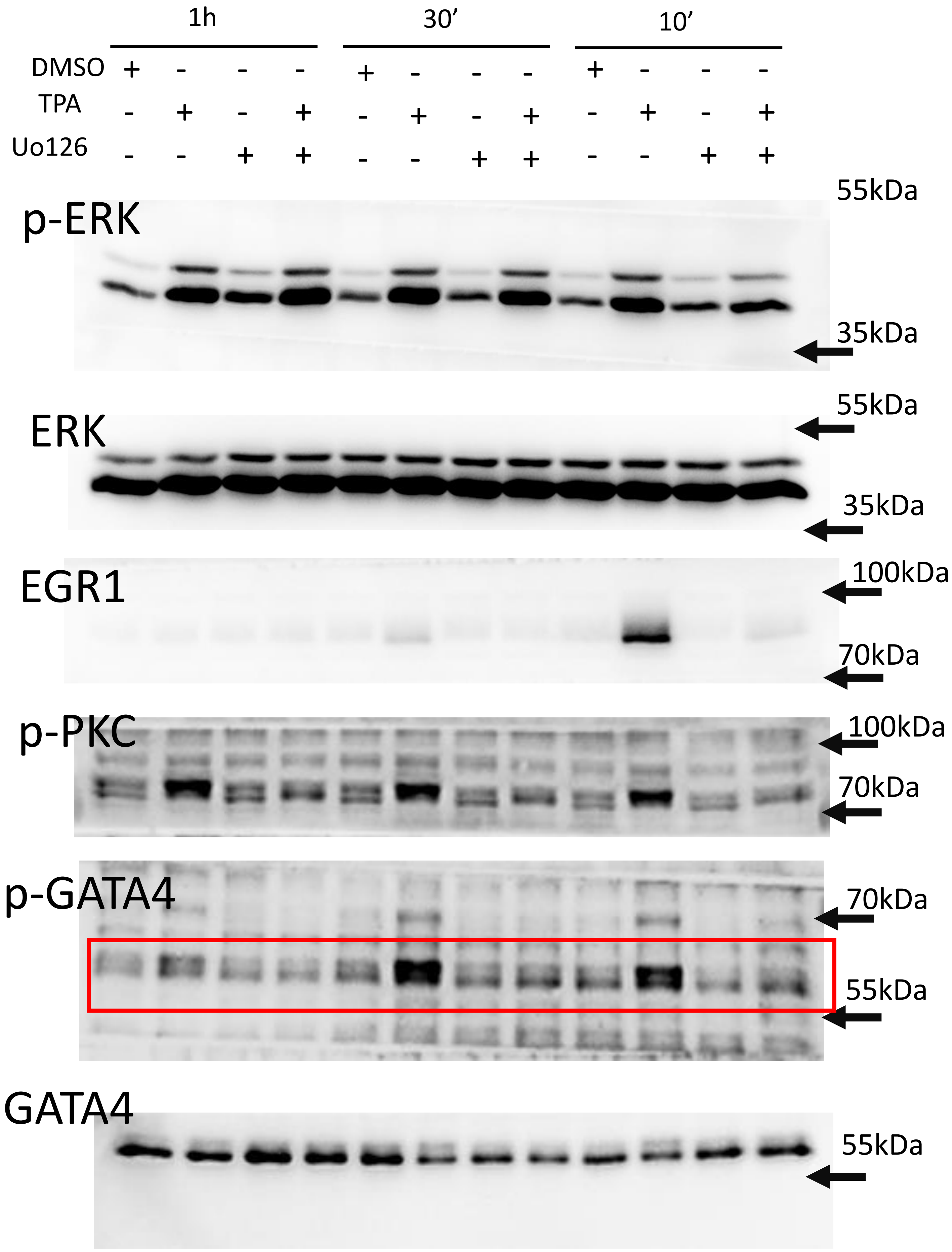

Supplementary Fig.15. Unprocessed Western blot membranes with molecular mass marker related to Fig. 6C-E. Bands surrounded by red frames are specific.

Supplementary Fig. 16

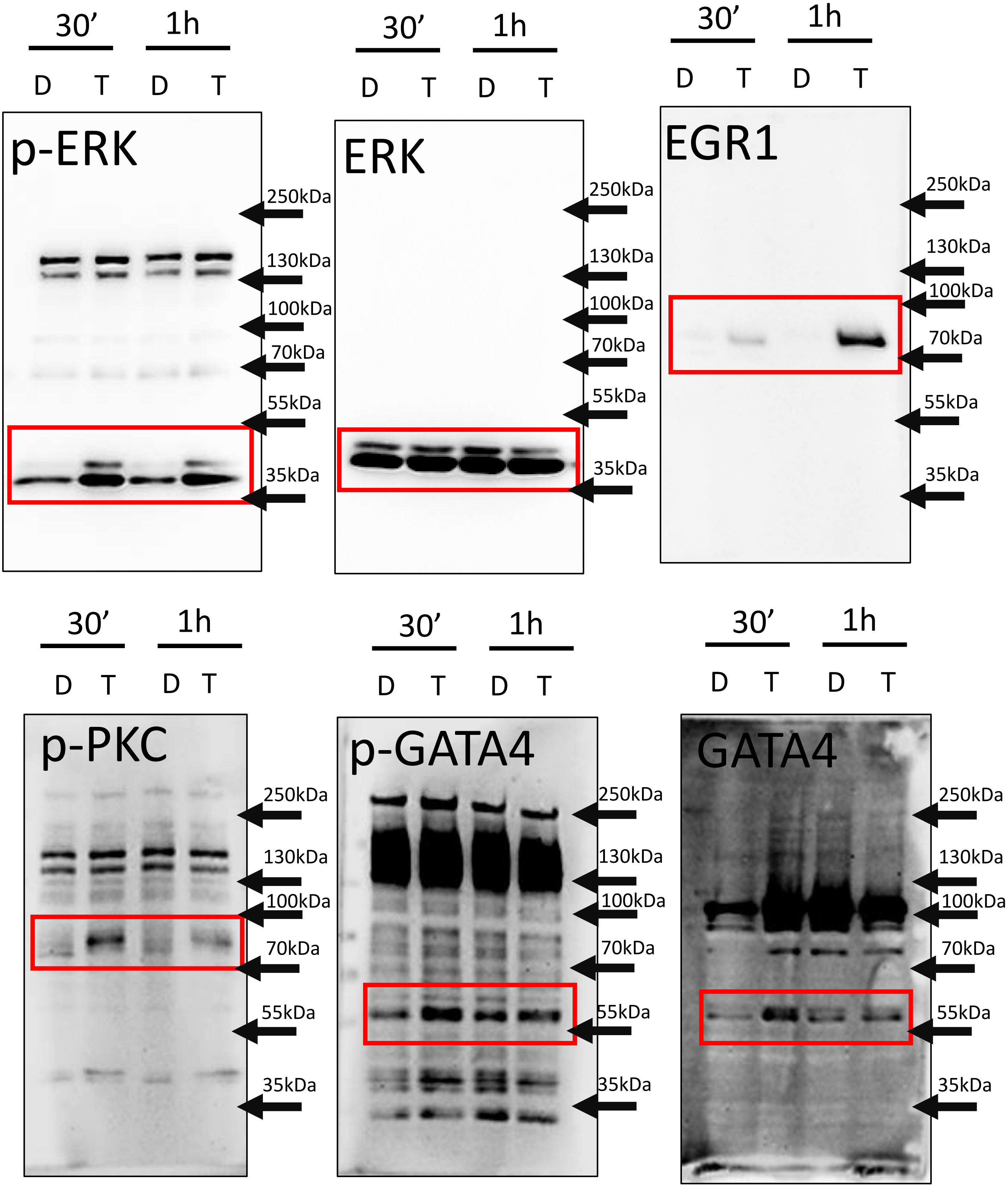

Supplementary Fig.16. Representative full-length Western blot membranes with molecular mass marker. R1 cell line-derived 6-day-old embryoid bodies were treated with 1 µM TPA (marked as T) and 0.1% DMSO (marked as D) in the indicated time intervals. Bands surrounded by red frames were taken as specific.

Supplementary Fig. 17

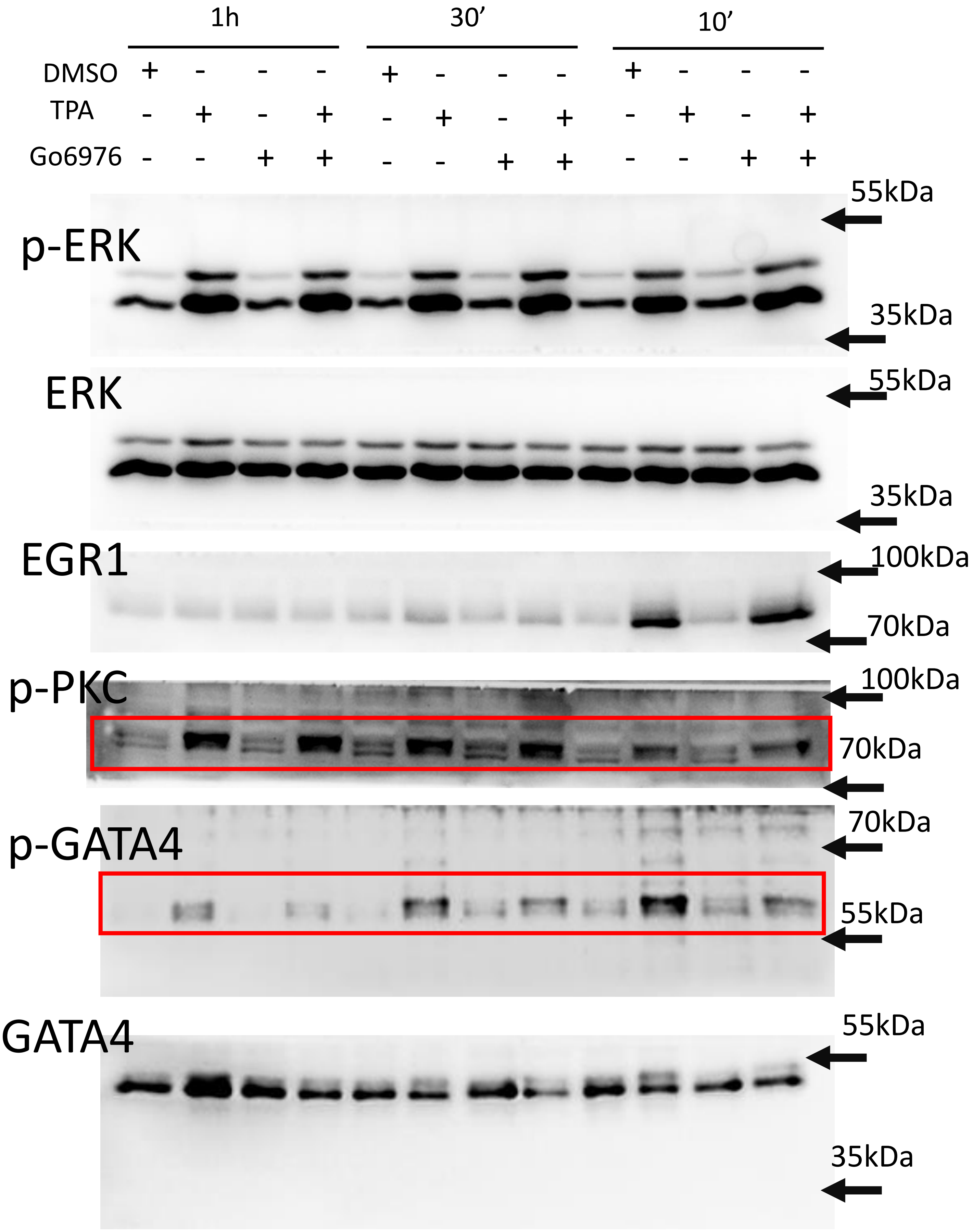

Supplementary Fig. 17

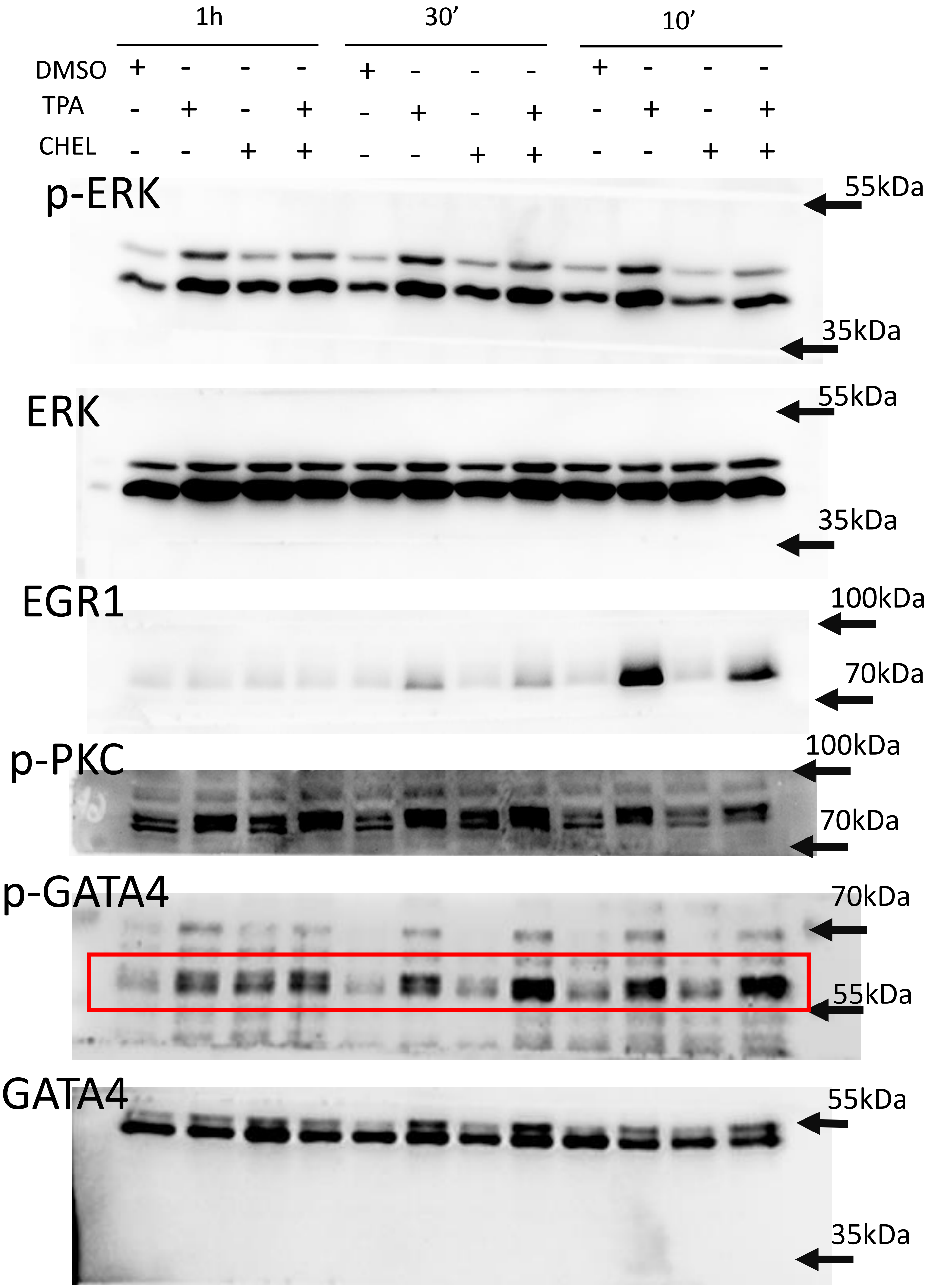

Supplementary Fig.17. Unprocessed Western blot membranes with molecular mass marker related to Supplementary Fig.7C,D. Bands surrounded by red frames are specific.

Supplementary Fig. 18

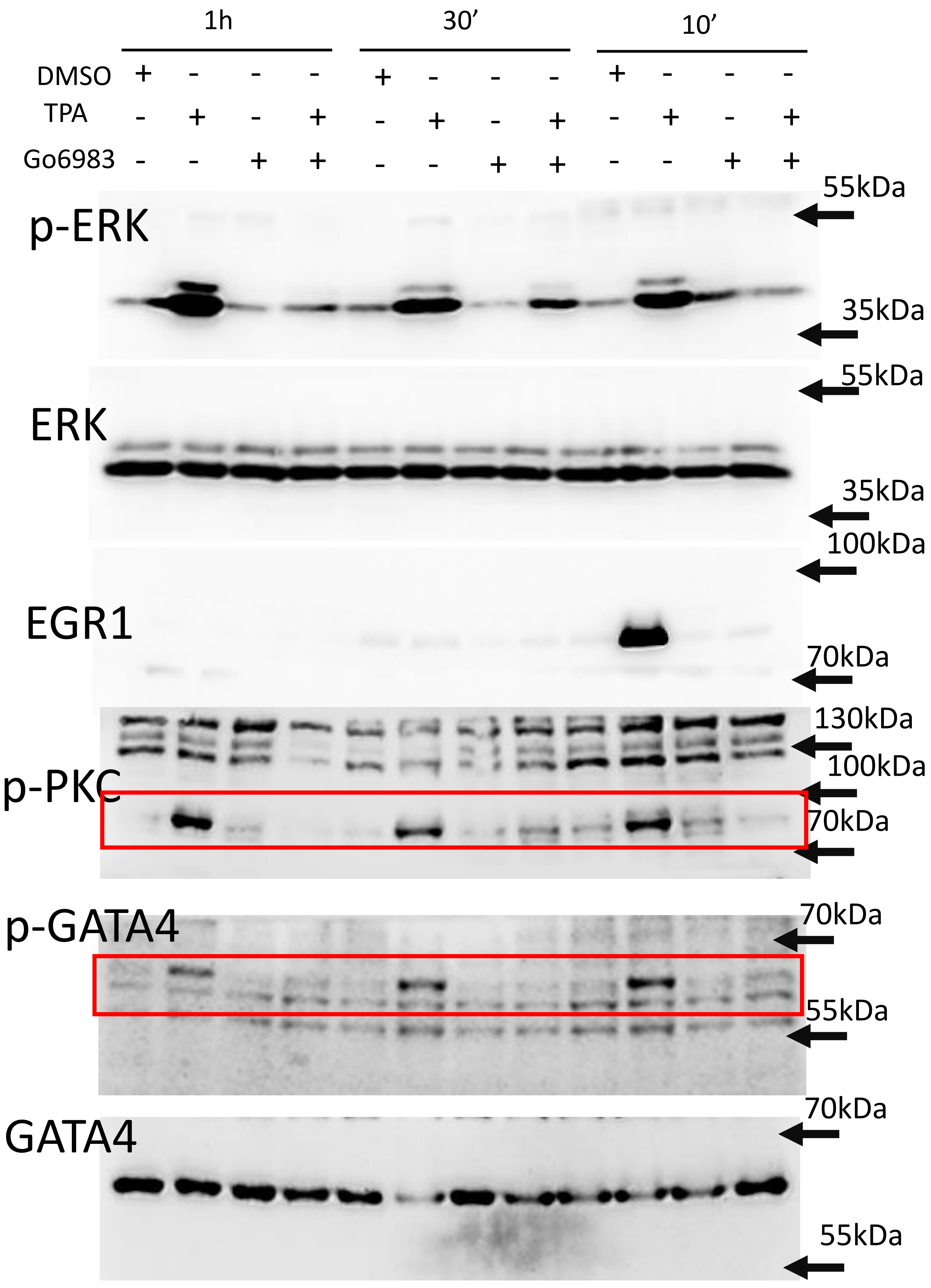

Supplementary Fig. 18

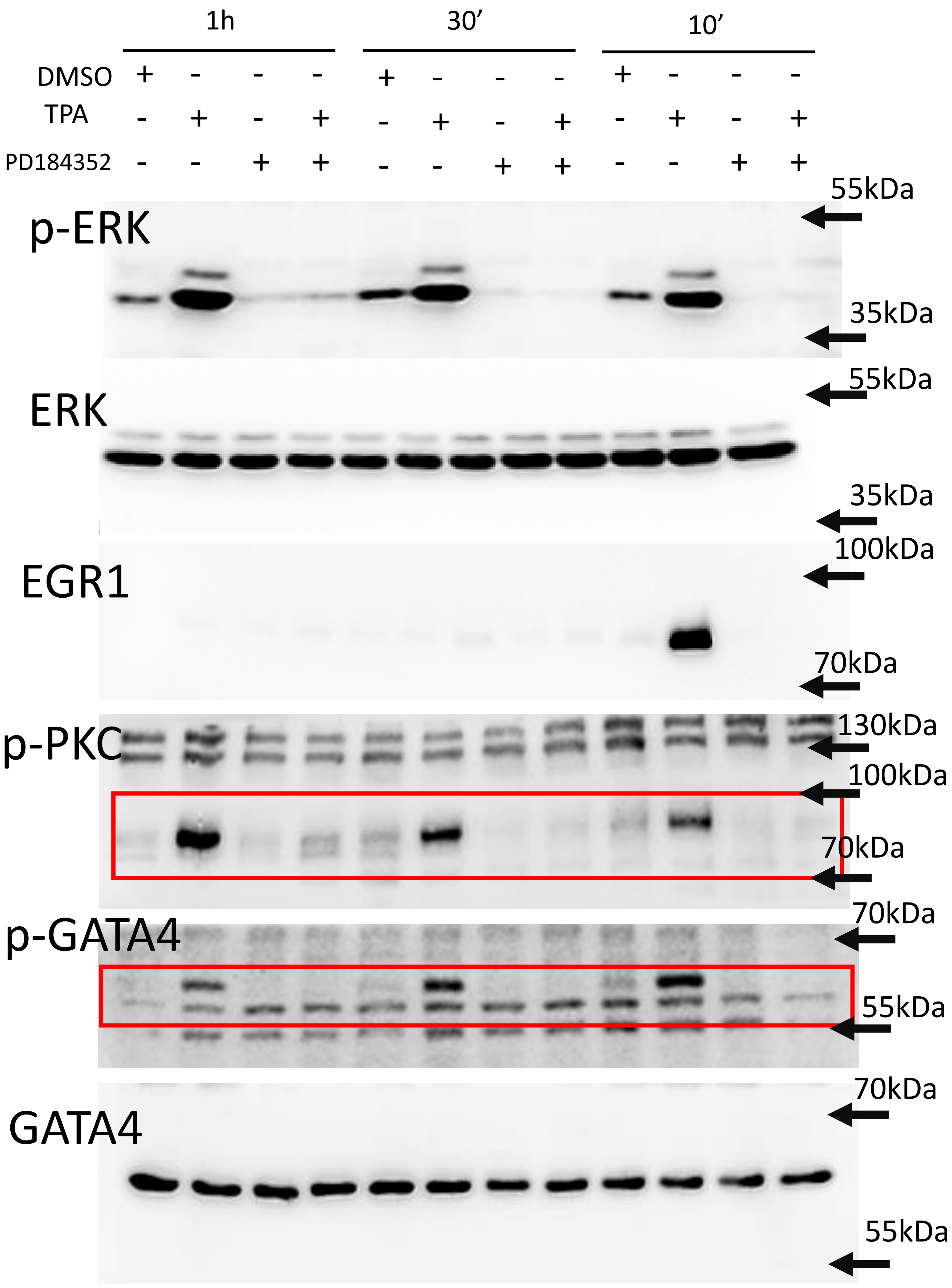

Supplementary Fig. 18

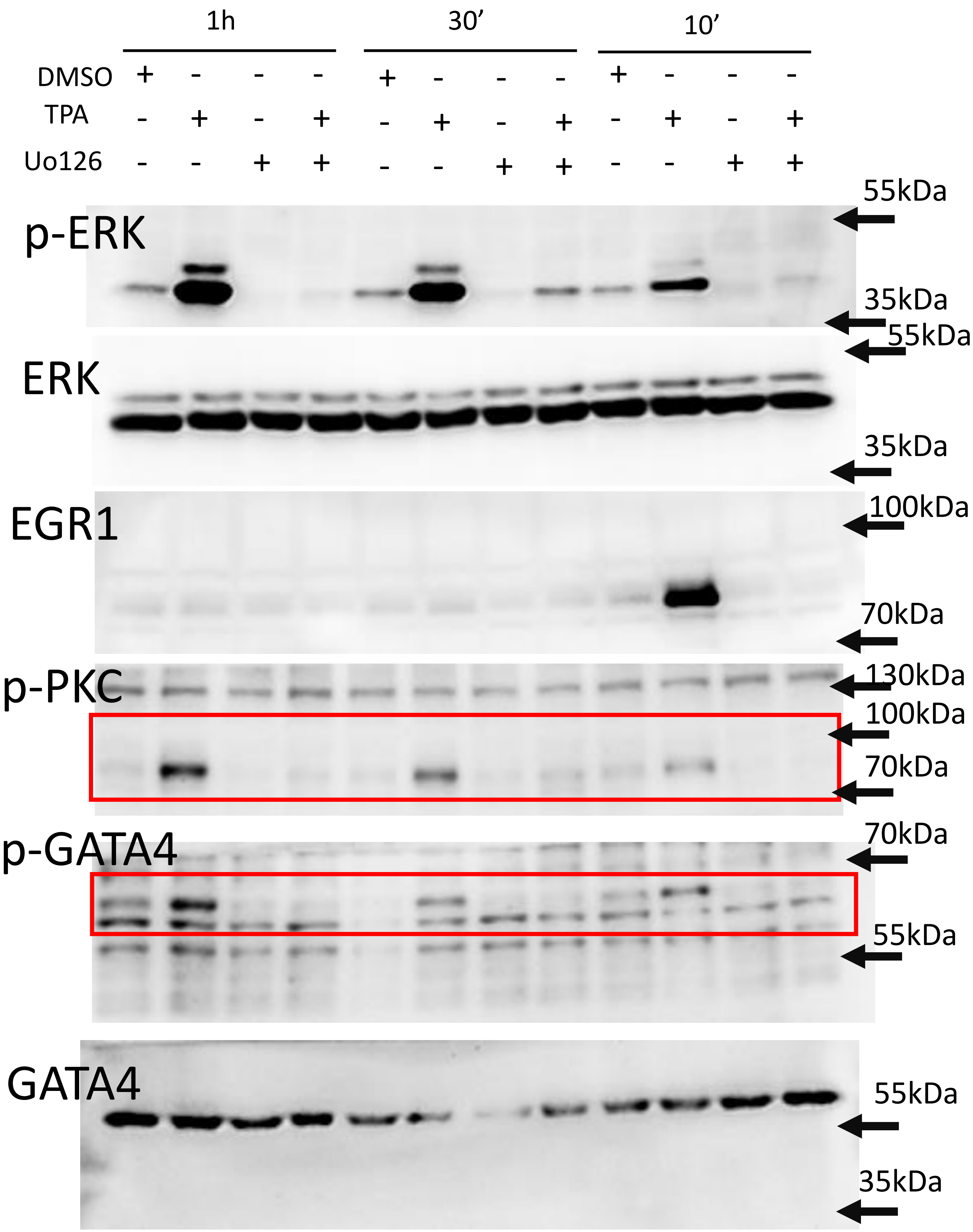

Supplementary Fig.18. Unprocessed Western blot membranes with molecular mass marker related to Supplementary Fig.8A-C. Bands surrounded by red frames are specific.

Supplementary Fig. 19

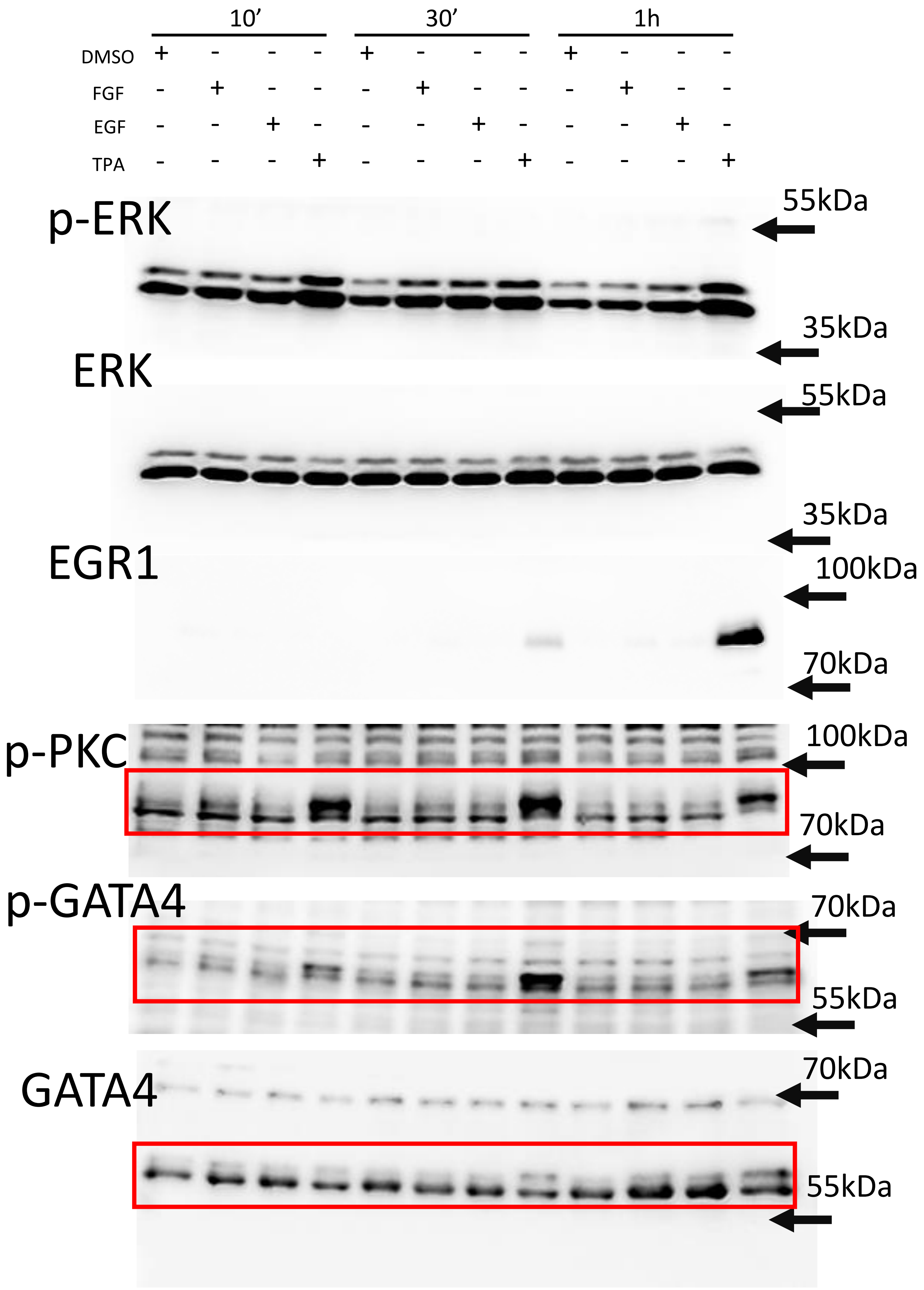

Supplementary Fig.19. Unprocessed Western blot membranes with molecular mass marker related to Supplementary Fig.11C. Bands surrounded by red frames are specific.
